# USP53 plays an antitumor role in hepatocellular carcinoma through deubiquitination of cytochrome c

**DOI:** 10.1038/s41389-022-00404-8

**Published:** 2022-06-02

**Authors:** Ye Yao, Weijie Ma, Yonghua Guo, Yingyi Liu, Peng Xia, Xiaoling Wu, Yiran Chen, Kunlei Wang, Chengjie Mei, Ganggang Wang, Xiaomian Li, Zhonglin Zhang, Xi Chen, Yufeng Yuan

**Affiliations:** 1 Department of Hepatobiliary and Pancreatic Surgery, Zhongnan Hospital of Wuhan University, Wuhan, 430071 Hubei China; 2 Minimally invasive treatment center of hepatobiliary and pancreatic diseases, Zhongnan Hospital of Wuhan University, Wuhan, 430071 Hubei P. R. China

**Keywords:** Oncogenes, Liver cancer

## Abstract

Despite of advances in treatment options, hepatocellular carcinoma (HCC) remains nearly incurable and has been recognized as the third leading cause of cancer-related deaths worldwide. As a deubiquitinating enzyme, the antitumor effect of ubiquitin-specific peptidase 53 (USP53) has been demonstrated on few malignancies. In this study, we investigated the potential antitumor role of USP53 in HCC. The results showed that USP53 was downregulated in HCC tissues as well as in HCC cell lines using both in silico data as well as patient samples. Furthermore, the ectopic expression of USP53 inhibited the proliferation, migration and invasion, and induced the apoptosis of HCC cells. Co-immunoprecipitation (CO-IP) assay and mass spectrometry (MS) combined with the gene set enrichment analysis (GSEA) identified cytochrome c (CYCS) as an interacting partner of USP53. USP53 overexpression increased the stability of CYCS in HCC cells following cycloheximide treatment. Finally, the overexpression of CYCS compensated for the decreased apoptotic rates in cells with USP53 knocked down, suggesting that USP53 induced the apoptosis in HCC cells through the deubiquitination of CYCS. To summarize, we identified USP53 as a tumor suppressor as well as a therapeutic target in HCC, providing novel insights into its pivotal role in cell apoptosis.

## Introduction

Hepatocellular carcinoma (HCC) accounted for 75–85% of primary liver cancer worldwide [1], and was reported to be the sixth most commonly diagnosed cancer and the third leading cause of cancer-related deaths worldwide in 2020 [1]. Similarly, in China, liver cancer ranked second in cancer-related mortality among both genders [2]. The major risk factors of HCC included chronic infection of hepatitis C virus (HCV), low load of hepatitis B virus (HBV) during treatment, and alcoholic or non-alcoholic fatty liver disease [3]. Currently, the major options for HCC treatment were consisted of surgical resection, radiofrequency ablation, transarterial chemoembolization, targeted therapy with tyrosine kinase inhibitors (TKIs) and immunotherapy with PD-1 or PD-L1 inhibitors [3, 4]. However, the prognosis of HCC patients remained poor with high recurrence rate. Therefore, it was urgent to identify novel therapeutic targets for HCC aiming to improve clinical outcomes.

Increasing studies suggested that the progression of HCC was associated with the function of ubiquitinating or deubiquitinating enzymes [5, 6]. Ubiquitin-specific proteases were deubiquitinating enzymes characterized by UBP type zinc finger domain and ubiquitin-specific protease domain. Initially identified in cholestasis [7], the tumor suppressive role of ubiquitin-specific peptidase 53 (*USP53*) had been reported in various cancers recently [8–10]. Zhao et al. showed that USP53 interacted with FKBP51 in lung adenocarcinoma cells, and induced apoptosis via the AKT pathway [8]. Furthermore, USP53 inhibited the progression of clear cell renal cell carcinoma by suppressing the nuclear factor κB (NF‐κB) pathway [9]. However, the potential role of *USP53* in HCC remained unknown. In this study, we explored the role of *USP53* as a tumor suppressor in HCC via the deubiquitination of cytochrome c (CYCS), which triggered the mitochondria apoptosis pathway.

## Materials and methods

### Cell lines

Five human liver cancer cell lines (HCCLM3, Huh-7, MHCC97H, MHCC97L, SMMC-7721), the normal liver cell line L02 and HEK-293T cell line were obtained from the Cell Bank of Shanghai Institute of Cell Biology (Chinese Academy of Medical Sciences, Shanghai, China). The cells were cultured in Dulbecco’s modified Eagle’s medium (DMEM, Gibco, CA, USA) supplemented with 10% of fetal bovine serum (Tico Europe, Netherlands) and 1% penicillin/streptomycin (Gibco, CA, USA) at 37 °C under 5% CO_2_. All cell lines were verified by short tandem repeat (STR) profiling and tested for mycoplasma contamination. The cells showed a distinct shape, even growth, clean and transparent intercellular substance, and a normal growth rate.

### Clinical specimens

A total of 33 pairs of HCC tumor and peri-tumor tissue samples were obtained from the Department of Hepatobiliary and Pancreatic Surgery of Zhongnan Hospital of Wuhan University (Wuhan, China). The written informed consent forms were obtained from the patients in this study. Paired HCC tumor tissues and adjacent normal tissues were resected from patients who were clinically and histologically diagnosed HCC without formerly chemotherapy or radiotherapy. Each specimen was split into three parts, and each part was preserved in RNAlater (Invitrogen, Carlsbad, CA, USA), homogenized in RIPA buffer (Biosharp, China) supplemented with 1% phenylmethanesulfonyl fluoride (PMSF, Biosharp, China), protease inhibitors and phosphatase inhibitor cocktail (Biosharp, Anhui, China), and fixed overnight in formaldehyde (Biosharp, China). All patients were regularly followed-up after the operation.

### Reagents

MG132 (HY-13259), chloroquine (CQ, HY-17589A), and BAX inhibitor peptide V5 (HY-P0081) were purchased from MCE (Shanghai, China). Cycloheximide (CHX, S7418) was purchased from Selleck Chemicals (Houston, TX, USA), while DMSO was obtained from Sigma (St. Louis, MO, USA, D2560). TUBE was obtained by LifeSensors (Pennsylvania, USA, UM-0302-0200).

### Quantitative real-time polymerase chain reaction (qRT-PCR)

RNA was extracted from the tissues and cells using TRIzol reagent (Invitrogen, USA). The concentration and purity of total RNA were measured using NanoDrop ND2000 (Thermo Scientific, Massachusetts, USA). Reverse transcription was performed using HiScript II Q RT SuperMix (Vazyme, Nanjing, China), and then the cDNA was amplified by qRT-PCR using the SYBR Green PCR kit (Vazyme, Nanjing, China) in the CFX96TM Real-Time System (Bio-Rad, California, USA). The relative gene expression levels were calculated by the comparative cycle threshold (Ct) method (2 − ΔCt) and normalized to that of β-actin. All assays were performed in triplicates. The primer sequences are listed in Supplementary Table S1.

### Western blotting

The lysates of Radio-Immunoprecipitation Assay (RIPA) buffer were centrifuged, and the protein content in the supernatants were measured using the BCA assay kit (Thermo Fisher Scientific, CA, USA). Equal amounts of protein per sample were separated by sodium dodecyl sulfate-polyacrylamide gel electrophoresis (SDS-PAGE) and then transferred onto a polyvinylidene fluoride membrane (Immobilon-P Transfer Membrane, Ireland). After blocked with 5% skim milk, the blots were incubated overnight with the primary antibodies at 4 °C, followed by the incubation of horseradish peroxidase-conjugated secondary antibody for 1 h at 37 °C. The positive bands were visualized using diaminobenzidine (DAB) substrate, and chemiluminescent signals were quantified using the Tanon-5200 (China) imager. The primary antibodies are listed in Supplementary Table S2.

### RNA interference

Small interfering RNA transfections were achieved by GenMute (SignaGen, Maryland, USA) in accordance with the manufacturer’s protocol. SiRNAs were synthesized by GENECREATE (Wuhan, China). The resulting constructs were verified by sequencing. The sequences were listed in Supplementary Table S3 and S4.

### Plasmid construction, lentiviral construction, and cell transfections

Flag-USP53 plasmid was amplified by PCR and cloned into phage-Flag vectors. The HA-CYCS plasmid, GST-HA plasmid, GST-HA-USP53 plasmid, GST-HA-CYCS plasmid, Flag-CYCS plasmid, and Flag-USP53-mut plasmid were provided by GENECREATE (Wuhan, China). To obtain Flag-USP53-mut plasmid, we conducted deletion mutation of the Cys-box of USP53(33-50LNEPGQNS CFLNSAVQVL), which is essential for the catalytic properties [11]. Ub-MYC plasmid was cloned as above. All constructs were verified by sequencing (TSINGKE, Beijing, China). The cells were transfected with the requisite plasmids using Lipofectamine 3000 (Invitrogen, USA) according to the manufacturer’s instructions. To obtain the USP53 lentivirus, HEK-293T cells were co-transfected with Flag-USP53 plasmid and lentiviral expression vectors using Lenti-PacTM HIV Expression Packaging kit (GeneCopoeia, USA) complying with the corresponding manufacturer’s protocol. Cells were harvested 48 h after transfection, and 1 ml of the lentivirus-containing medium was used to infect 1 × 10^6^ HCC cells.

### Establishment of in vivo HCC models

Six-weeks-old male Balb/c nude mice (*n* = 10) were purchased from SPF Biotechnology (Beijing, China), and housed at the Central Laboratory of Animal Science in Wuhan University with ambient temperature and humidity. Then, the mice were randomly allocated into either the experimental group or the control group (5 mice/group) and were injected subcutaneously with 5 × 10^6^ control or USP53-overexpressing HCCLM3 cells in 100 μl DMEM into their right armpits. After 4 weeks, the mice were euthanized, and the subcutaneous tumors were dissected for further analysis. To establish orthotopic tumors, the mice were anesthetized and injected intra-hepatically with 1 × 10^6^ HCC cells by laparotomy. Positron emission tomography-computed tomography (PET-CT) was conducted after four weeks. The investigators were blinded to group allocation when assessing the outcomes. All animal experiments were approved by the animal ethics committee of Wuhan University’s Institutional Animal Care and Use Committee of Center, and in accordance with the guidelines of the National Institute of Health guide for the care and use of Laboratory animals (NIH Publications No. 8023, revised 1978).

### Immunohistochemistry (IHC)

The clinical samples as well as the xenograft tissues were fixed in paraformaldehyde, embedded in paraffin, and cut into 4 μm-thick sections. IHC was performed using the UltraSensitive^TM^ S-P kit (Maixin, Fuzhou, China) as per the manufacturer’s protocol. The antibodies were listed in Supplementary Table S2.

We scored the percentage of IHC positive cells based on the following five categories (0–4): 0 (< 5%), 1 (5–25%), 2 (26–50%), 3 (51–75%), and 4 (>75%). Four categories (0–3): 0 (negative), 1 (weakly positive), 2 (moderately positive), and 3 (strongly positive) were used to grade the staining intensity of the stained cells. The two scores were multiplied to calculate the final staining index. The staining assessments were performed by two independent pathologists.

### Transwell assay

HCC cells were seeded in the upper of Matrigel-coated transwell chambers (pore size, 8 μm) (BD Biosciences, USA) in serum-free media at the density of 1 × 10^4^ cells/well in a 24-well plate. The lower chambers were filled with complete DMEM. After cultured for 48 h, the chambers were fixed with formaldehyde and stained with crystal violet. The number of cells on the lower surface of the insert were counted.

### Wound-healing assay

The appropriately treated cells were seeded in 6-well plates and cultured till they were fully confluent. The monolayer was scratched longitudinally with a sterile pipette (100 μl). After the dislodged cells were washed off, the serum-free DMEM was added. Cells were cultured for 72 h, and then the wound region was photographed at 0, 24, 48, and 72 h to measure the gap width, respectively.

### EdU staining

Cells in the logarithmic growth phase were incubated with EdU solution, and then stained with an EdU staining kit of KeyGEN (KeyGEN BioTECH, Nanjing, China) according to the manufacturer’s instructions.

### Colony-formation assay

Cells were seeded in a 6-well plate at the density of 10^3^ cells per well in complete DMEM, and cultured for 2–3 weeks. The ensuing colonies were fixed with formaldehyde, stained with crystal violet, and counted.

### Cell Counting Kit-8 (CCK8) assay

Cells were plated in 96-well plates in 100 μl DMEM, and cultured for varying durations. A total of 10 µl CCK8 reagent (Biosharp, Anhui, China) was added into the cells at the stipulated time points, followed by the incubation of 2 h. The absorbance at 450 nm was measured using a microplate reader (Thermo MULTISCAN FC, USA).

### Analysis of cell apoptosis and cell cycle

Annexin V-fluorescein isothiocyanate (FITC)/propidium iodide (PI) kit (MULTI SCIENCE, Hangzhou, China) and cell cycle staining kit (MULTI SCIENCE, Hangzhou, China) were used to analyze the apoptotic rates and cell cycle profiles, respectively. The percentage of apoptotic cells and the distribution of cells in different phases of cell cycle were analyzed by flow cytometry (FC500 flow cytometer, Beckman-Coulter, USA).

### TUNEL assay

We performed TUNEL assay (TUNEL Assay Kit – FITC, Abcam, Cambridge, UK; #ab66108) based on the manufacturer’s instructions.

### Caspase-3 activity

To evaluate the activity of caspase-3, we used a caspase-3 activity kit (Beyotime Institute of Biotechnology, Haimen, China). After the designated treatments were administered, we prepared cell lysates, which were then incubated with a reaction buffer containing the caspase-3 substrate (Ac-DEVD-pNA) (2 mM) on 96-well microtiter plates. The reaction systems were incubated at 37 °C for 8 h. Absorbance of the samples was measured at 405 nm using an ELISA reader. The detailed analysis procedure was performed by following the manufacturer’s instructions.

### Co-immunoprecipitation (CO-IP), mass spectrometry (MS) and silver staining

HEK-293T cells were seeded in 10 cm tissue culture dishes, transfected with Flag-USP53 plasmid, and lysed with 1 ml IP buffer (20 mM Tris-HCl, pH 7.4; 150 mM NaCl, 1 mM EDTA pH 8, 1% NP-40, 1× Protease and Phosphatase Inhibitor Cocktail). The cell lysis was rotated and mixed 30 min before pre-washed Protein A/G Magnetic beads (MedChemExpress, Shanghai, China) and anti-flag antibody or IgG were added and then incubated upside down for more than 3 h at 4 °C. Finally, the interaction protein was pulled down by the magnetic beads. The magnetic beads were resuspended in SDS-loading buffer and then heated up to 96 °C for 10 min before loaded to polyacrylamide gel. Silver staining assay was conducted by Celerity Silver Staining Kit (Beyotime Biotechnology, Shanghai, China) according to the manufacturer’s instructions. Subsequently, gels were used for MS-based analysis. For Exogenous Co-IP, HA-CYCS and Flag-USP53 plasmids were co-transfected into HEK-293T cells. The following protocols were the same as above.

### Ubiquitination assays and Tandem Ubiquitin Binding Entity

For the ubiquitination assays, HEK-293T, Huh-7, and HCCLM3 cells were co-transfected with Myc-Ub, HA-CYCS, Flag-USP53, or Flag-USP53-mut plasmids and were lysed using an IP lysis buffer. The following steps were the same as the CO-IP. An anti-Myc antibody was used to detect the ubiquitination of HA-CYCS. For the CYCS poly-ubiquitination study, Huh-7 cells were starved for 4 h before being treated with a starvation medium containing MG132 for an additional 4 h. The cell lysates were immunoprecipitated using an anti-CYCS antibody (Abcam) and were immunoblotted with TUBE (high affinity ubiquitin binding peptide) for 8 h and detected in the same way as WB. The antibodies and dilution ratio used are listed in Supplementary Table S2.

### Immunofluorescence (IF)

The suitably treated cells were plated and fixed on coverslips, permeabilized with 0.5% Nonidet P-40 (NP-40) for 20 min, and blocked with 5% bovine serum albumin (BSA) for 30 min. After incubated with the primary antibodies (Supplementary Table S2) for 2 h in room temperature, the samples were probed with fluorescein-conjugated secondary antibody for 1 h and counterstained with 4',6-diamino-2-phenyl-indole (DAPI). The slides were observed under a confocal microscope (Leica, Wetzlar, Germany).

### Proximity ligation assay

A Duolink® proximity ligation assay kit (Merck, DUO92102, Darmstadt, Germany) was used to perform the proximity ligation assay by following the manufacturer’s instructions. Anti-USP53 Rabbit polyclonal Ab (Thermo Fisher, PA5-57424) was conjugated with a PLUS nucleotide probe, while anti-CYCS mouse monoclonal Ab was conjugated with a MINUS nucleotide probe. All images were visualized and captured using an Olympus Fluoview 1000 laser scanning confocal microscope (Leica, Wetzlar, Germany). The MitoTracker was purchased from Beyotime (C1048, Shanghai, China), while DAPI was obtained from Servicebio (G1012, Wuhan, China).

### GST pull-down

For GST pull-down analysis, the full-length sequence of USP53 was cloned into pGEX-6P-1 containing the GST tag. The plasmids of GST-HA-USP53 and Flag-CYCS were transfected into *E.coli*. Fusion proteins GST-HA-USP53 and GST-HA were purified. About 100 µg of GST-HA and GST-HA-USP53 fusion protein were mixed with 50 µL of glutathione agarose, and mixed upside down at 4 °C for 60 min. After 3 washes with PBST, approximately 100 µg of Flag-CYCS fusion protein was added to immobilized GST-HA-USP53 and GST-HA. The two fusion proteins were incubated overnight at 4 °C with gentle rotation. Bound proteins were eluted with elution buffer (10 mM glutathione in PBS, pH 8.0) and analyzed by immunoblotting. Correspondingly, the binding of purified GST-HA-CYCS fusion protein with Flag-USP53 was verified following the same method as above.

### Isolation and extraction of cytoplasm and mitochondria

The cells were washed using PBS and then incubated on ice for 30 min with 300 µl of a lysis buffer (68 mM sucrose, 200 mM mannitol, 50 mM KCl, 1 mM EGTA, 1 mM EDTA, 1 mM DTT, and 1×Complete protease inhibitor (Boehringer Mannheim)). After homogenization using a Dounce homogenizer (using a B-type pestle), the cells were centrifuged at 4 °C (800 g). The supernatant was transferred into a new tube to be centrifuged at 14,000 g for 10 min. The supernatant (cytosol) and pellets (mitochondria) were stored at –80 °C for IB analysis.

### GSEA analysis

GO analyses were conducted using gene set enrichment analysis (GSEA). GO gene sets was analyzed using the *c5.all.v7.1symbols.gmt*. We loaded the list of all the names of the genes and corresponding quantitative value. The number of permutation was set to be 1000. We divided the samples into USP53 high and low expression groups according to the median. The false discovery rate (FDR) value was set at <0.05.

### Kaplan–Meier plotter analysis

The RNA-seq data of USP53 and the corresponding clinical information of HCC patients were extracted from The Cancer Genome Atlas (TCGA). The disease-specific survival (DSS) and overall survival (OS) of HCC patients were plotted by the Kaplan–Meier Plotter (http://www.kmplot.com). The cutoff value was automatically selected in a systematic manner according to the ranking of USP53 expression level [12].

### Statistical analysis

GraphPad Prism 8.0 software and SPSS 21.0 were used for statistical analysis. All data were expressed as the mean ± standard deviation (SD), and analyzed using the Student’s *t*-test and Spearman’s correlation analysis as appropriate. *P* < 0.05 was considered to be statistically significant.

## Results

### *USP53* was downregulated in HCC tissues and correlated with poor prognosis

**Fig. 1:**
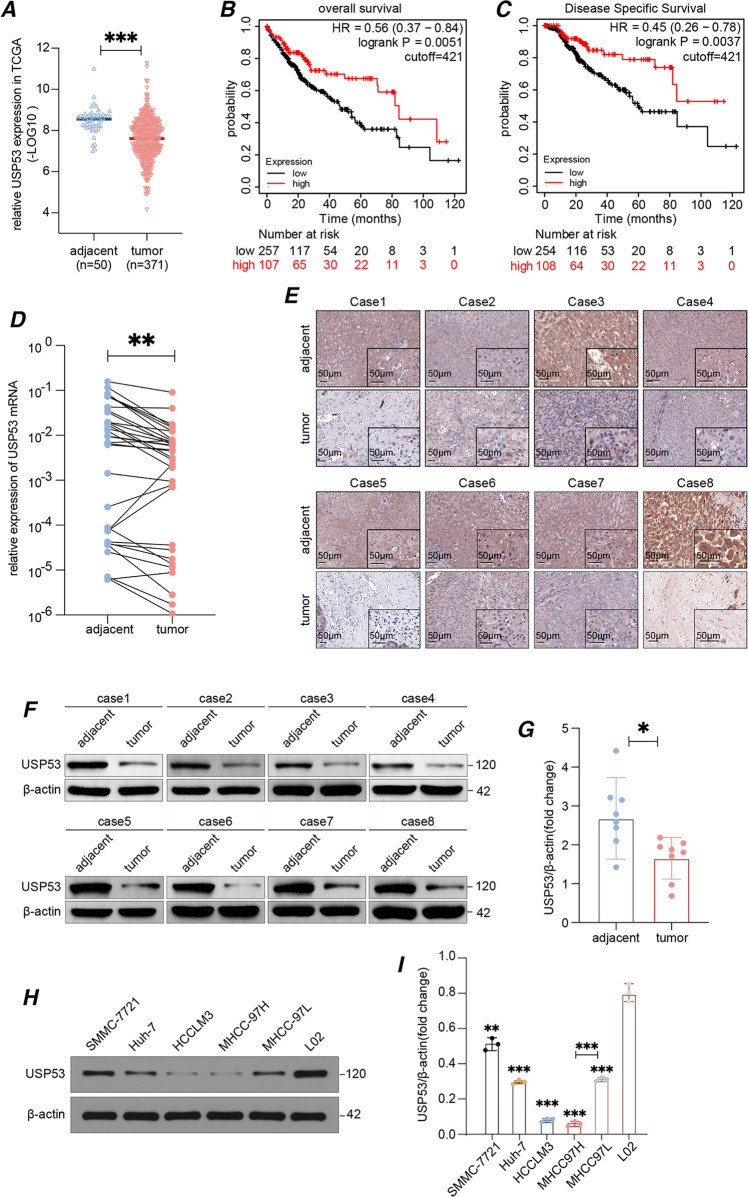
USP53 was downregulated in HCC and was related to the prognosis of HCC. **A** USP53 was downregulated in 371 HCC tissues compared to 50 adjacent non-tumor tissues based on TCGA-LIHC dataset. The data was (-LOG10) for USP53 expression(fpkm). The line showed the average value. **B**, **C** Kaplan–Meier curves showing the OS and DSS rates of USP53^high^ and USP53^low^ groups. The curves were plotted by Kaplan–Meier Plotter (http://www.kmplot.com). The data was DEseq normalized**. D** RT-qPCR of USP53 mRNA expression in 33 pairs of HCC and adjacent tissues. **E** Representative IHC images showing in-situ expression of USP53 in eight pairs of HCC and adjacent non-tumor tissues (magnification 100x and 400x). Scale bar: 50 μm. **F**, **G** Immunoblot showing USP53 protein levels in eight pairs of HCC and adjacent peri-tumor tissues. **H**, **I** USP53 proteins levels in SMMC-7721, Huh-7, HCCLM3, MHCC97H, MHCC97L, and L02 cell lines. Statistical analysis was based on the difference in the expression levels of HCC cells and L02. *p < 0.05; **p < 0.01; ***p < 0.001.

In order to assess the role of *USP53* in the development of HCC, we evaluated its expression levels in 371 HCC samples and 50 peri-tumor samples using the RNA-seq data from TCGA-LIHC database. As shown in Fig. 1A, *USP53* mRNA levels were significantly reduced in the tumor tissues compared to the adjacent liver tissues. The patients were divided into the *USP53*^high^ and *USP53*^low^ groups based on the median expression level, and the rates of OS and DSS were significantly higher in patients with high expression of *USP53* (Fig. 1B, C). To verify these results, we analyzed USP53 mRNA and protein expression levels in 33 and eight pairs of HCC tumor and peri-tumor tissues using RT-qPCR, IHC, and WB assays. USP53 showed a significant decrease at both mRNA (Fig. 1D) and protein (Fig. 1E–G) level in the tumor tissues compared with the adjacent tissue. Consistent with this, multiple HCC cell lines also expressed lower levels of the *USP53* protein compared to the normal liver cell line L02 (Fig. 1H, I). Furthermore, the MHCC97H cell line showed a lower level of USP53 expression compared with MHCC97L expression, with MHCC97H being a more malignant cell line based on a previous finding [13].

### Overexpression of USP53 inhibited the proliferation and migration of HCC cells in vitro

**Fig. 2:**
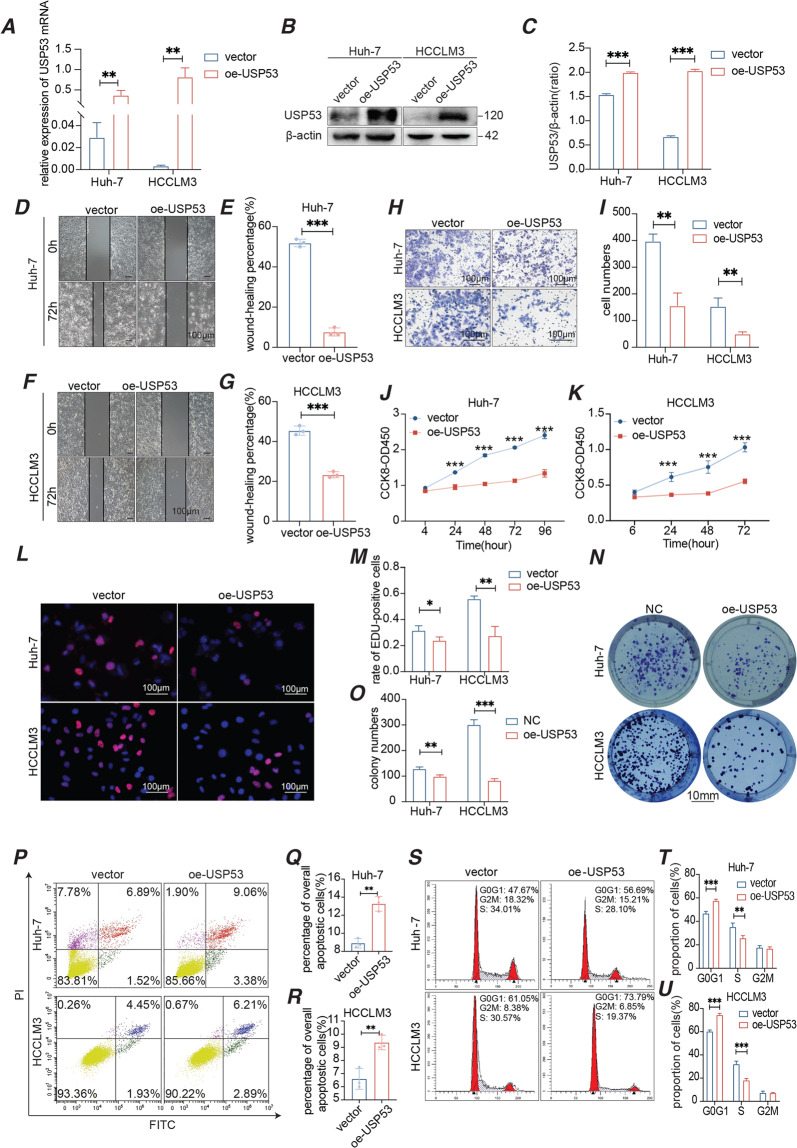
Overexpression of USP53 inhibited the growth of HCC cells in vitro. **A**–**C** USP53 mRNA and protein levels in Huh-7 and HCCLM3 cell lines in which USP53 overexpression plasmid was suitably transfected. **D**–**G** Representative images of the wound-healing assay showing migration distance of the control and USP53-overexpressing cells. Magnification 100x. Scale bar:100 μm. The wound-healing percentage was calculated by the distance changes between the two sides. **H**, **I** Representative images of the trans-well assay showing invasion capacity of control and USP53-overexpressing cells. Scale bar:100 μm. **J**, **K** Percentage of the proliferating cells in the indicated groups. There were 2000 cells per group initially. *n* = 6. **L**, **M** Representative images showing EdU uptake in the control and USP53-overexpressing Huh-7 and HCCLM3 cells. EdU was marked by red fluorescent dyes. DAPI which stained the nucleus showed the blue fluorescence. Magnification 400x. Scale bar:100 μm. **N**, **O** Number of colonies formed by the control and USP53-overexpressing Huh-7 and HCCLM3 cells. Scale bar: 10 mm. **P**–**R** Flow cytometry plots showing percentage of apoptotic control and USP53-overexpressing Huh-7 and HCCLM3 cells. The cells were marked by annexin V. **S**–**U** Flow cytometry plots showing the cell cycle distribution of control and USP53-overexpressing Huh-7 and HCCLM3 cells. All experiments were repeated three times. *p < 0.05; **p < 0.01; ***p < 0.001.

To further explore the function of USP53 in HCC, we ectopically expressed the gene in Huh-7 and HCCLM3 cell lines, and confirmed its overexpression at the mRNA and protein levels (Fig. 2A–C). Wound-healing and transwell assays showed that the overexpressed USP53 significantly inhibited the migration (Fig. 2D–G) and invasion (Fig. 2H, I) of HCC cells in vitro. Furthermore, Huh-7 and HCCLM3 cells with overexpressed USP53 had significantly weakened the proliferative capacity of HCC cells (Fig. 2J, K) and showed lower uptake of EdU (Fig. 2L, M), indicating there were fewer cells in the S phase. Consistent with this, USP53 overexpression markedly decreased the number and size of the colonies formed by HCC cells in vitro (Fig. 2N, O). In addition, the apoptotic rates were significantly higher in the USP53-overexpressing cells compared to the controls (Fig. 2P–R). Finally, USP53 overexpression led to an increase in the proportion of cells in the G1 phase, and a concomitant decrease of that in the S phase (Fig. 2S–U). Taken together, USP53 inhibited the proliferation and migration of HCC cells by inducing the blocking at G1/S phases and increasing cell apoptosis.

### Overexpression of USP53 reduced tumor growth in vivo

**Fig. 3:**
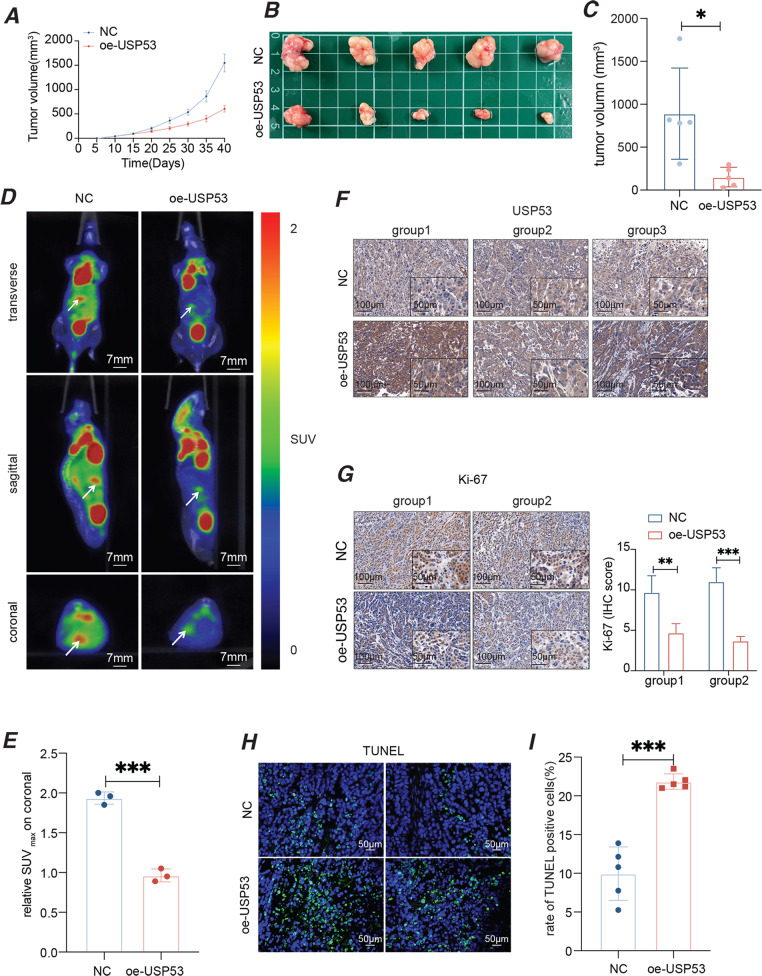
Overexpression of USP53 inhibited HCC xenograft growth in vivo. **A** Tumor growth kinetics curve of oe-USP53 group and control. *n* = 5. **B** Tumors obtained from nude mice that implanted with oe-USP53 HCCLM3 cells (*n* = 5) and control HCCLM3 cells (*n* = 5). **C** Tumor volume in the control and USP53-overexpression groups. *n* = 5. **D**, **E** PET-CT images of orthotopic HCC xenografts in the indicated groups. *n* = 3. **F** Representative images showing the in-situ expression of USP53 in three pairs of xenograft tissues. Magnification 100x and 400x. **G** Representative images showing the in-situ expression of Ki-67 in the tumor xenografts of NC group and USP53-overexpression groups. Magnification 100x and 400x. **H**, **I** Representative images showing TUNEL + cells in the indicated xenografts. Magnification 400x. All experiments were repeated three times. *p < 0.05; **p < 0.01; ***p < 0.001.

To validate the findings in vitro, we established the xenografts of the control as well as HCCLM3 cells with USP53 stably overexpressed in a mouse model. As shown in Fig. 3A–C, USP53 overexpression significantly decreased the growth rate and the tumor volume, which implied lower proliferative ability. Furthermore, orthotopic tumors derived from the USP53-overexpressing HCC cells showed weaker PET-CT signals compared to control group, which were derived from the same HCCLM3 cells without USP53 overexpression (Fig. 3D, E). The in-situ expression of USP53 in the tumor tissues was verified by IHC (Fig. 3F). In addition, USP53 overexpression significantly decreased the percentage of Ki-67+ proliferating cells and increased that of TUNEL + apoptotic cells in the tumor tissues compared to the control (Fig. 3G–I). Taken together, USP53 inhibited HCC growth in vivo by impairing cell proliferation and enhancing cell apoptosis.

### USP53 upregulated CYCS and its downstream genes through direct interaction

**Fig. 4:**
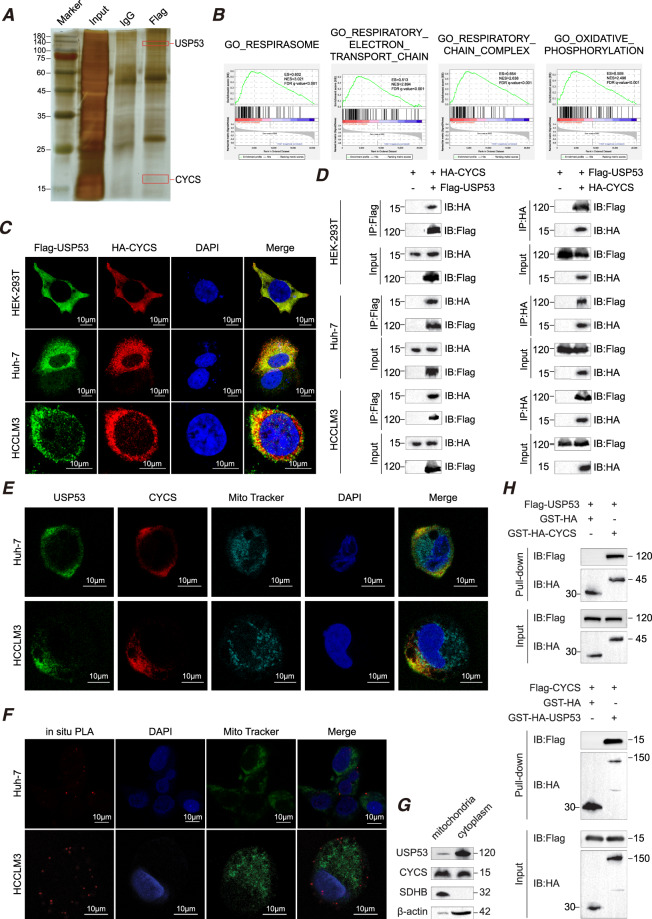
CYCS directly interacts with USP53. **A** Representative images of silver-stained protein bands and mass spectrometry (MS) analysis. The result was obtained after three times’ repeat. **B** Gene signature associated with CYCS in the USP53^low^ and USP53^high^ groups of TCGA-LIHC dataset as identified by GSEA. Three hundred seventy-one samples were divided into USP53^low^ and USP53^high^ groups according to the median of USP53 expression level. Number of permutations:1000. **C** Representative exogenous immunofluorescence images showing the co-localization of USP53 and CYCS. Scale bar:10 μm. **D** Exogenous co-immunoprecipitation showing interaction of USP53 and CYCS. The results were repeated three times. **E** Endogenous immunofluorescence images showing the co-localization of USP53 and CYCS and their position relationship with mitochondria (stained by MitoTracker) in Huh-7 and HCCLM3 cells. Scale bar:10 μm. **F** Proximity ligation assay (PLA) showed the direct connection of USP53 and CYCS, which takes place outside of mitochondria. Scale bar: 10 μm. **G** Mitochondrial and cytoplasmic proteins were isolated and detected separately, revealed the subcellular location of USP53 were mostly in cytoplasmic in Huh-7 cell line. The experiment was repeated three times. **H** GST pull-down assay showed the direct binding of USP53 and CYCS in HEK-293T cells. Pure GST was used as control. The experiment was repeated three times.

To explore the mechanisms underlying the inhibitory effect of USP53 in HCC, we identified the proteins that directly interacted with USP53 in the HCC cells by CO-IP/MS (Supplementary Table S5, Fig. 4A). The possible pathways involved in USP53’s function were then identified from TCGA database (https://portal.gdc.cancer.gov/) by gene set enrichment analysis (GSEA) using GSEA software 4.0.1. We downloaded the TCGA-LIHC data and divided it into two groups based on USP53 expression level and analyzed it using GSEA. As shown in Fig. 4B, respirasome, respiratory electron transport chain, respiratory chain complex and oxidative phosphorylation were significantly enriched. CYCS is a mitochondrial protein [14, 15] that induced the apoptotic cascade through recruiting the apaf-1 and caspase-9 to generate the active apoptosome and indirectly via respiratory failure when released into the cytoplasm [16]. Various studies had demonstrated that CYCS could induce apoptosis in many different kinds of malignant cells [17–23]. IF (Fig. 4C) and CO-IP (Fig. 4D) experiments showed that USP53 co-localized with CYCS in HEK-293T, Huh-7 and HCCLM3 cells. To further investigate the subcellular location of USP53 and CYCS, we determined endogenous immunofluorescence using the mitochondrial marker, MitoTracker. The results showed that USP53 and CYCS shared the same location outside of mitochondria (Fig. 4E), which was in line with the results of the Proximity Ligation Assay (Fig. 4F). Furthermore, we isolated mitochondrial and cytoplasmic proteins and performed WB detection. The results showed that USP53 was almost entirely located in the cytoplasm, while CYCS was located both in the mitochondria and cytoplasm (Fig. 4G). SDHB and β-actin were utilized as a reference for the mitochondria and cytoplasm, respectively. Finally, GST pull-down assay was used to verify the direct interaction between USP53 and CYCS (Fig. 4H).

**Fig. 5:**
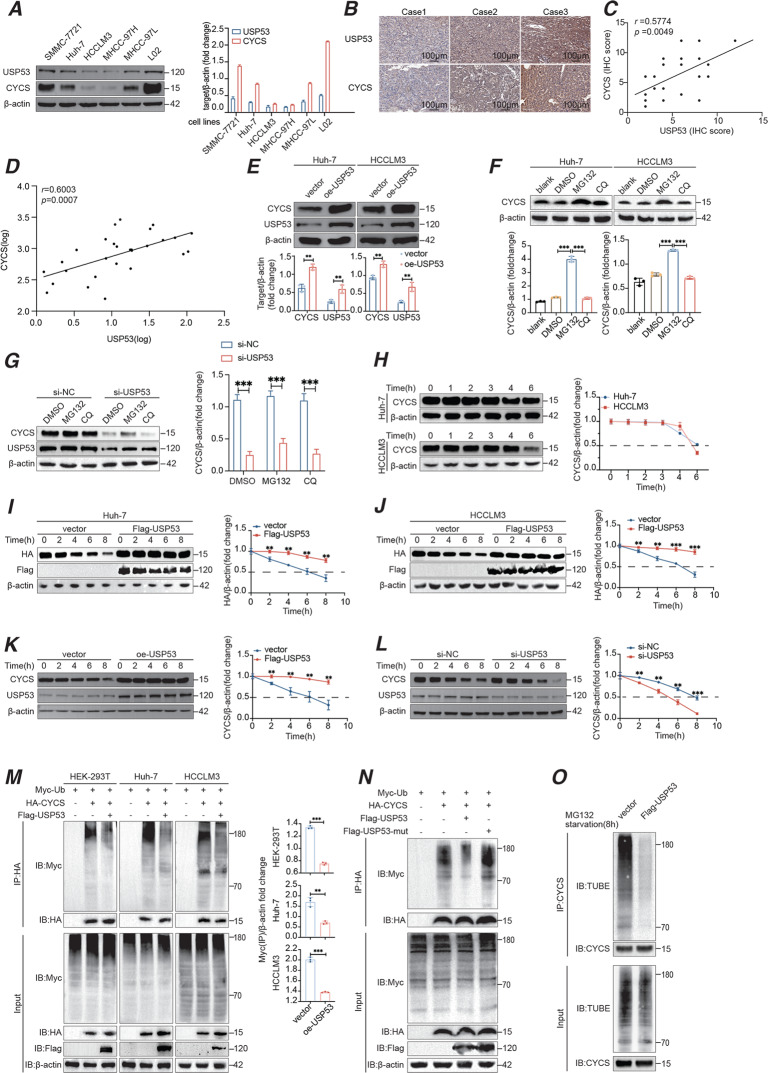
USP53 induced deubiquitination of CYCS and enhanced apoptosis. **A** Western bolt analysis of USP53 and CYCS proteins in HCC cell lines and L02 hepatocytes showing their relation of expression level in cell lines. **B** IHC staining of USP53 and CYCS in patient tissues. Scale bar:100 μm. **C** Spearman’s correlation analysis of USP53 and CYCS based on the IHC assay of patient samples. (*n* = 21). **D** Spearman correlation analysis of USP53 and CYCS mRNA level (LOG) in clinical samples of HCC tissues. (*n* = 28). **E** CYCS levels in USP53-overexpressing Huh-7 and HCCLM3 cells. **F** Western blot analysis of CYCS protein in Huh-7 and HCCLM3 cells. Cells were treated with 25 μM MG132 or CQ for 8 h before harvest. **G** Western blot analysis of CYCS after USP53 was knocked down in Huh-7 cells, the cells were treated with 25 μM MG132 or CQ for 8 h before harvest. **H** Western blot analysis showing the protein degradation time of CYCS in Huh-7 and HCCLM3 cells. **I**, **J** Western blot analysis of HA-CYCS protein level in Huh-7 and HCCLM3 cells transfected with HA-tagged CYCS plasmids and Flag-tagged USP53 plasmids or empty vectors. The transfected cells were treated with 100 μg/ml cycloheximide (CHX) for indicated time points. The results were normalized. **K**, **L** Western blot analysis of endogenous CYCS protein in Huh-7 cells with USP53 overexpressed or knocked down. The transfected cells were treated with 100 μg/ml cycloheximide (CHX) for indicated durations. Each group were repeated for three times. The results were normalized. **M** IP assays in HEK-293T cells, Huh-7 cells, and HCCLM3 cells showing that USP53 overexpression resulted in the deubiquitination of CYCS. IP assays were conducted 48 h after all the plasmids co-transfected. **N** IP analysis of the ubiquitination of HA-tagged CYCS in Huh-7 cells transfected with mutant USP53 and Flag-tagged USP53. **O** Western blotting of CYCS poly-ubiquitination. Cells were starved for 4 h before treated with starvation medium containing MG132 for additional 4 h. Cell lysates were immunoprecipitated by anti-CYCS antibody and immunoblotted with TUBE (high affinity ubiquitin binding peptide) following the same method of WB. All experiments were repeated three times. *p < 0.05; **p < 0.01; ***p < 0.001.

To assess the regulation function of USP53 and CYCS, we detected their mRNA and protein level expression in cell lines and clinical samples. As shown in Fig. 5A, the CYCS protein expression showed a similar trend to that of USP53 in HCC cells and L02. IHC conducted on 21 HCC patients showed a positive correlation (Fig. 5B–C), while the RT-qPCR results also revealed the positive relationship between *USP53* and *CYCS* mRNA levels in tissues (Fig. 5D).

As reported previously, USP53 induced the apoptosis of lung adenocarcinoma cells through deubiquitination of FKBP51 [8]. Based on the fact that CYCS can be degraded by the ubiquitination-proteasome pathway [24, 25], we hypothesized that USP53 regulated CYCS through a similar mechanism. As shown in Fig. 5E, CYCS was upregulated in the cells overexpressing USP53. However, the CYCS mRNA level didn’t change with the USP53’s trend (Supplementary Fig. 1). In addition, MG132 could significantly decrease the degradation speed of CYCS under both normal conditions and when USP53 was knocked down compared with the CQ group and the control group (Fig. 5F, G). Basing on the results of our previous experiments, the half-life of CYCS was around 3–4 h in the Huh-7 cells and 4–6 h in HCCLM3 cells (Fig. 5H). In the presence of the protein translation inhibitor, cycloheximide, USP53 overexpression in HCC cells increases the stability of CYCS, both endogenously and exogenously (Fig. 5I–K). On the contrary, USP53 - caused the acceleration of CYCS degradation (Fig. 5L). Ubiquitination CO-IP further showed that overexpression of USP53 led to deubiquitination of CYCS in the HEK-293T, Huh-7 and HCCLM3 cells (Fig. 5M). However, when the catalytic site was muted, the deubiquitination effect of USP53 overexpression on CYCS disappeared (Fig. 5N). Then, we used Tandem Ubiquitin Binding Entities (TUBE) to blot the CYCS immunoprecipitation samples with USP53 overexpression and control cells in the Huh-7 cell line. Cell lysates (Input) showed similar ubiquitination degree, however, the immunoprecipitated CYCS revealed a significantly decrease in USP53 overexpressed cells (Fig. 5O). Taken together, USP53 enhanced the stability of CYCS in HCC cells by blocking the ubiquitination and the subsequent degradation.

### USP53 induced apoptosis in HCC cells through stabilization of CYCS

**Fig. 6:**
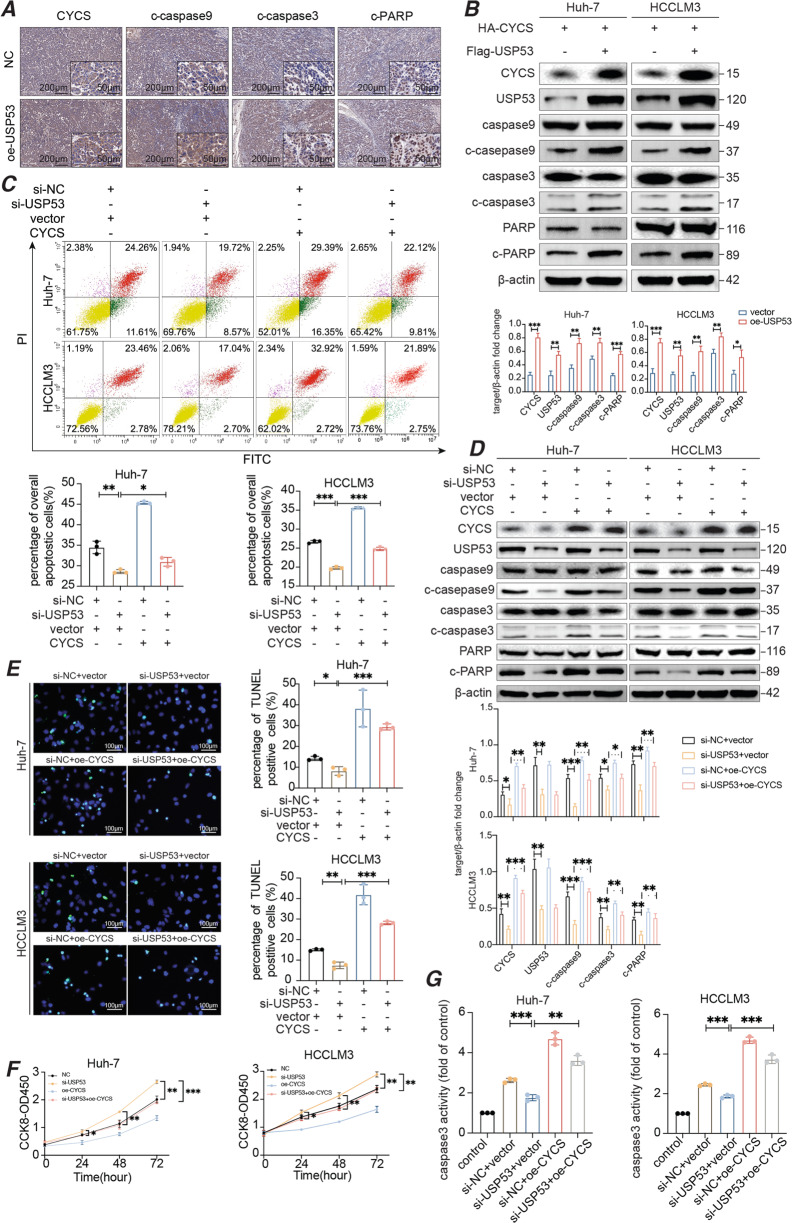
USP53 induced apoptosis via deubiquitination of CYCS. **A** Immunohistochemical staining of CYCS, c-caspase-9, c-caspase-3, c-PARP in xenograft tumors from the USP53 overexpression and control group. (magnification 100x and 400x). **B** Immunoblot showing the expression of apoptosis-related proteins in the indicated groups. **C** Flow cytometry plots showing the apoptotic rates in HCC cells co-transfected with si-USP53 and HA-CYCS or control. *n* = 3. **D** Immunoblot showing the expression of apoptosis-related proteins in CYCS-overexpressing and USP53-knockdown cells. **E** Representative images showing TUNEL + cells after CYCS overexpression and USP53 knockdown. Magnification: 400x. Scale bar:100 μm. **F** CCK8 assay revealed proliferation capacity of the indicated groups. **G** Caspase-3 activity of the indicated groups of Huh-7 and HCCLM3 cells. Data are described with means ± SD of the three independent experiments. Each value is expressed as the fold of caspase-3 activation level to the control level, and the value of control was set to 1. All experiments were repeated three times. *p < 0.05; **p < 0.01; ***p < 0.001.

The upregulation of CYCS in the USP53-overexpressing cells was accompanied by increased cell apoptosis as well as higher expression levels of the pro-apoptotic cleaved-caspase-9, cleaved-caspase-3 and cleaved-PARP proteins, which was observed both in the sections of subcutaneous tumor tissues and in HCC cells (Fig. 6A, B). The upregulation of CYCS expression in the USP53 overexpressed xenografts was detected using WB (Supplementary Fig. 2). As shown in Fig. 6B, to induce a more obvious effect of USP53 overexpression on CYCS and its downstream apoptotic molecules, we transfected the cells in the two groups with the same amount of HA-CYCS plasmids to create an apoptotic environment. To further confirm whether USP53 induced cell apoptosis through CYCS, we simultaneously knocked down USP53 and overexpressed CYCS in HCC cells (Supplementary Fig. 3), and found the apoptotic rate was significantly enhanced compared to knocking down USP53 alone (Fig. 6C). CYCS overexpression restored the levels of the pro-apoptotic proteins in the USP53-knockdown cells (Fig. 6D). Furthermore, cells overexpressing CYCS showed higher apoptotic rates (Fig. 6E), lower proliferative capacity (Fig. 6F), and higher caspase-3 activity (Fig. 6G), compared with cells with *USP53* silencing. On the contrary, the knockdown of CYCS expression (Supplementary Fig. 3) when USP53 was overexpressed revealed the same result (Supplementary Fig. 4A–J). Similarly, the overexpression of USP53 exerted a similar effect on the BAX inhibitor (Supplementary Fig. 4K–T). These findings indicated that the tumor suppressive role of USP53 might be facilitated by CYCS.

According to the results above, we summarized the mechanism of the whole study. As shown in Supplementary Fig. 5, USP53 interacted with CYCS and promotes the deubiquitination and stabilization of CYCS in HCC cells, which further activated the apoptosis cascade and inhibits the progression of tumors.

## Discussion

Studies increasingly showed that ubiquitination and deubiquitination of key proteins were crucial for the process of oncogenesis [26, 27]. The role of deubiquitinating enzyme USP39 had been demonstrated in the progression of hepatocellular carcinoma [28]. Besides, USP53 could also induce cell apoptosis in lung adenocarcinoma cells through deubiquitinating FKBP51 [8]. In addition, USP53 also inhibited the proliferation and migration of clear renal cell carcinoma cells by inactivating the NF-κB pathway [9]. We found that USP53 was significantly downregulated in HCC tissues and cell lines compared to their normal counterparts. Furthermore, forced expression of USP53 inhibited the proliferation, migration and invasion, and induced apoptosis in HCC cell lines both in vitro and in vivo. We identified the key apoptotic protein CYCS as a binding partner of USP53 in HCC cells. Consistent with its deubiquitinating function, USP53 stabilized CYCS in HCC cells by preventing ubiquitination and subsequent degradation, which increased the levels of the downstream apoptotic proteins. Furthermore, ectopic expression of CYCS in HCC cells enhanced apoptotic rates and even compensated for USP53 silencing, indicating that CYCS might be a crucial mediator of the antitumor effects of USP53.

CYCS usually resided in the space between the outer and inner membranes of mitochondria [29, 30], and could be released into the cytoplasm in response to signals of cell death and activated by the pro-apoptotic Bcl2-family of proteins [31, 32]. In the cytoplasm, CYCS interacted with the caspases and triggers the apoptotic cascade [33–35]. In addition, the release of CYCS could also lead to the respiratory failure and increase the generation of reactive oxygen species in the mitochondria, resulting in oxidative stress and cell death [35], which was consistent with the observed association between USP53 overexpression and respirasome, respiratory electron transport chain, respiratory chain complex, and oxidative phosphorylation in GSEA. However, it remained to be elucidated whether USP53 could regulate oxidative phosphorylation as well as the underlying mechanisms. According to THE HUMAN PROTEIN ATLAS (https://www.proteinatlas.org/), USP53 was mainly localized to plasma membranes and the cytoplasm. The experimental data obtained in this study also demonstrated that the binding between USP53 and CYCS occurred in the cytoplasm. However, it remained unclear whether USP53 had an impact on the release of CYCS into the cytosol. Furthermore, our experimental data showed that USP53 interacts with the CYCS protein. Interestingly, there was a positive correlation between the mRNA levels of *USP53* and *CYCS* in clinically obtained tissues, but the cell experiments did not show a similar result. We speculated that there might be some complex feedback mechanisms that cause indirect long-term regulation in the tissue that cause the mRNA levels of *USP53* and *CYCS* to be correlated, while the cellular level observations 2–3 days after the experiment did not reveal a similar result. Moreover, according to UbiBrowser (http://ubibrowser.ncpsb.org.cn/), CYCS had multiple sites for ubiquitination, and the potential E3 ligases had also been predicted (Supplementary Table S6). Therefore, we hypothesized that USP53 regulated the balance between the ubiquitination and deubiquitination of CYCS through the competition for the binding site of E3 on CYCS. Our study will focus on this speculation and potential related mechanisms.

## Conclusion

USP53 enhanced the apoptosis of HCC cells via deubiquitination of CYCS, and had a potential as a promising novel therapeutic target for HCC.

## Supplementary information

supplementary table S1-S6

supplementary figure1

supplementary figure2

supplementary figure3

supplementary figure4

supplementary figure5

legend of supplementary figures

statement for changing the order of author

language editorial certificate

## Data Availability

The data that support the findings of this study are available from the corresponding author upon reasonable requests.
