# Correction: USP53 plays an antitumor role in hepatocellular carcinoma through deubiquitination of cytochrome c

**DOI:** 10.1038/s41389-023-00470-6

**Published:** 2023-05-15

**Authors:** Ye Yao, Weijie Ma, Yonghua Guo, Yingyi Liu, Peng Xia, Xiaoling Wu, Yiran Chen, Kunlei Wang, Chengjie Mei, Ganggang Wang, Xiaomian Li, Zhonglin Zhang, Xi Chen, Yufeng Yuan

**Affiliations:** 1 Department of Hepatobiliary and Pancreatic Surgery, Zhongnan Hospital of Wuhan University, Wuhan, 430071 Hubei China; 2 Minimally invasive treatment center of hepatobiliary and pancreatic diseases, Zhongnan Hospital of Wuhan University, Wuhan, 430071 Hubei P. R. China

**Keywords:** Oncogenes, Liver cancer

Correction to: *Oncogenesis* **11**, 31 (2022) 10.1038/s41389-022-00404-8, published online 02 June 2022

The graph of cell apoptosis of HCCLM3 cell line detected by Flow cytometry in Figure 6c erroneously made the 4th group a duplication of the 2nd group. The original and corrected figure is provided below. This mistake was made unconsciously during figure preparation and did not alter the result or conclusion in any way. The data used in the bar scale was in line with the original data downloaded for the Flow cytometry machine, which was correct version.

The authors sincerely apologize to the editors and readers for this error and any inconvenience it may cause.

Original figure:
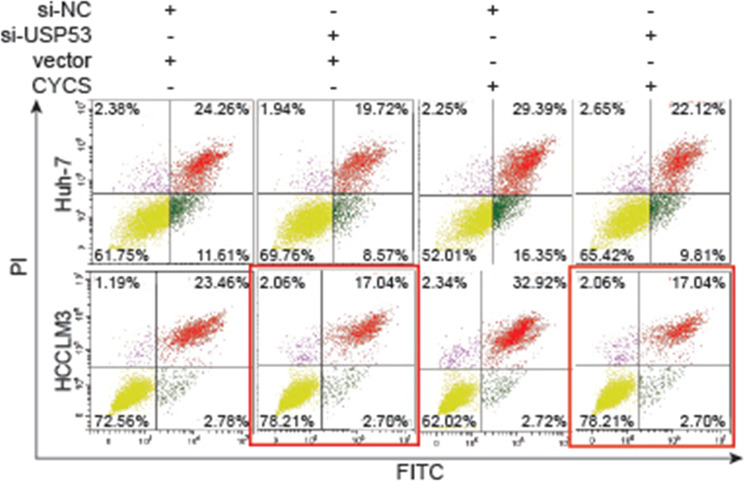


Corrected figure:
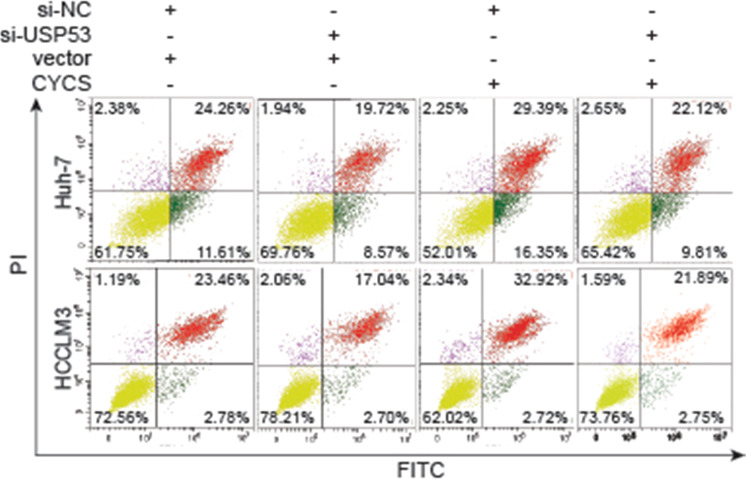


The original article has been corrected.

