# Supplementary material for: USP53 plays an antitumor role in hepatocellular carcinoma through deubiquitination of cytochrome c
Source: Oncogenesis. 2022 Jun 2;11(1):31. doi: 10.1038/s41389-022-00404-8 (PMC9163188; doi:10.1038/s41389-022-00404-8)
Supplement: Supplementary file 1 — supplementary table S1-S6 [file 41389_2022_404_MOESM1_ESM.docx]

| Gene name | | Forward primer | Reverse primer |
| --- | --- | --- | --- |
| β-actin | CATGTACGTTGCTATCCAGGC | | CTCCTTAATGTCACGCACGAT |
| USP53  CYCS | CTCAGGAATTTGGAAGCAGGC  GAGCGGGAGTGTTCGTTGTG | | TCTTGCATTATGGTCCTTGGGT  CTTCCGCCCAAAGAGACCAT |

Supplementary Table S1. Sequences of primers used in q-PCR analysis

Supplementary Table S2. Primary antibodies used in this study

| Antigens | Manufacturer | Catalog Number | application |
| --- | --- | --- | --- |
| USP53  USP53  USP53 | ABclonal  Thermo Fisher  SUSABIO | A14353  PA5-57424  CSB-PA030132 | 1:3000 for WB  1:200 for IF  1:200 for IHC |
| β-actin | ABclonal | AC026 | 1:10000 for WB |
| CYCS  CYCS | Proteintech Group  Abcam | 10993-1-AP  Ab110325 | 1:1000 for WB  1:200 for IF |
| Caspase 9 | Proteintech Group | 10380-1-AP | 1:1000 for WB; 1:100 for IHC |
| Caspase 3 | Cell signaling technology | #9662 | 1:1000 for WB |
| Cleaved caspase3 | Cell signaling technology | #9664 | 1:1000 for WB; 1:2000 for IHC |
| PARP | Cell signaling technology | #9532 | 1:1000 for WB |
| Cleaved-PARP | Cell signaling technology | #5625 | 1:1000 for WB; 1:50 for IHC |
| Flag-tag | Cell signaling technology | #14793 | 1:1000 for WB; 1:800 for IF |
| Flag-tag | Cell signaling technology | AE005 | 1:100for IP |
| HA-tag | MBL | M180-3 | 1:150 for IP; 1:1000 for IF |
| HA-tag | Cell signaling technology | #3724 | 1:1000 for WB |
| Myc-tag | MBL | M047-3 | 1:1000 for WB |
| IgG  SDHB  TUBE | Proteintech Group  HUABIO  lifesensors | B900610  ER1803-63  UM-0302-0200 | 1:100 for IP  1:1000 for WB  1:1000 for WB |

Supplementary Table S3. Sequences of siRNA against USP53 used in this study

| Name | Sequence |
| --- | --- |
| siRNA NC | 5’-UUCUCCGAACGUGUCACGU-3’ |
| siRNA #1 | 5’-CACUUCCCUCAGAUAACAUAATT-3’ |
| siRNA #2 | 5’-CCUGUAAUUCAUAAGUCAGAUTT-3’ |
| siRNA #3 | 5’-GCAGCCAAGAUUCUAGGGAUATT-3’ |

Supplementary Table S4. Sequences of siRNA against CYCS used in this study

| Name | Sequence |
| --- | --- |
| siRNA NC | 5’-UUCUCCGAACGUGUCACGU-3’ |
| siRNA #1 | 5’-CCAAAUCUCCAUGGUCUCUUUTT- 3’ |
| siRNA #2 | 5’-CCAAGAAGUACAUCCCUGGAATT-3’ |
| siRNA #3 | 5’-GCAUUAAGAAGAAGGAAGAAATT-3’ |

Supplementary Table S5: List of candidates USP53 binding proteins from MS analysis

| num | protein description | ΣCoverage | | Σ# Proteins | Σ# Unique Peptides | Σ# Peptides | Σ# PSMs | # AAs | MW [kDa] | calc. pI |
| --- | --- | --- | --- | --- | --- | --- | --- | --- | --- | --- |
| 1 | Acetyl-CoA carboxylase 1 OS=Homo sapiens OX=9606 GN=ACACA PE=1 SV=2 - [ACACA_HUMAN] | | 13.90 | 1 | 16 | 18 | 22 | 2346 | 265.4 | 6.37 |
| 2 | Keratin, type I cytoskeletal 18 OS=Homo sapiens OX=9606 GN=KRT18 PE=1 SV=2 - [K1C18_HUMAN] | | 45.81 | 1 | 15 | 15 | 22 | 430 | 48.0 | 5.45 |
| 3 | Keratin, type II cytoskeletal 8 OS=Homo sapiens OX=9606 GN=KRT8 PE=1 SV=7 - [K2C8_HUMAN] | | 28.99 | 5 | 12 | 13 | 18 | 483 | 53.7 | 5.59 |
| 4 | Methylcrotonoyl-CoA carboxylase subunit alpha, mitochondrial OS=Homo sapiens OX=9606 GN=MCCC1 PE=1 SV=3 - [MCCA_HUMAN] | | 22.21 | 1 | 9 | 9 | 13 | 725 | 80.4 | 7.78 |
| 5 | RNA-binding protein 28 OS=Homo sapiens OX=9606 GN=RBM28 PE=1 SV=3 - [RBM28_HUMAN] | | 15.02 | 1 | 9 | 9 | 16 | 759 | 85.7 | 9.22 |
| 6 | Pyruvate carboxylase, mitochondrial OS=Homo sapiens OX=9606 GN=PC PE=1 SV=2 - [PYC_HUMAN] | | 8.49 | 1 | 6 | 6 | 8 | 1178 | 129.6 | 6.84 |
| 7 | ATP-dependent RNA helicase DDX18 OS=Homo sapiens OX=9606 GN=DDX18 PE=1 SV=2 - [DDX18_HUMAN] | | 13.13 | 1 | 5 | 5 | 6 | 670 | 75.4 | 9.50 |
| 8 | Propionyl-CoA carboxylase alpha chain, mitochondrial OS=Homo sapiens OX=9606 GN=PCCA PE=1 SV=4 - [PCCA_HUMAN] | | 8.93 | 1 | 5 | 5 | 6 | 728 | 80.0 | 7.52 |
| 9 | Heterogeneous nuclear ribonucleoprotein A1 OS=Homo sapiens OX=9606 GN=HNRNPA1 PE=1 SV=5 - [ROA1_HUMAN] | | 16.67 | 2 | 4 | 4 | 4 | 372 | 38.7 | 9.13 |
| 10 | C-1-tetrahydrofolate synthase, cytoplasmic OS=Homo sapiens OX=9606 GN=MTHFD1 PE=1 SV=3 - [C1TC_HUMAN] | | 7.91 | 1 | 4 | 4 | 4 | 935 | 101.5 | 7.30 |
| 11 | Proliferation marker protein Ki-67 OS=Homo sapiens OX=9606 GN=MKI67 PE=1 SV=2 - [KI67_HUMAN] | | 2.64 | 1 | 4 | 4 | 5 | 3256 | 358.5 | 9.45 |
| 12 | T-complex protein 1 subunit alpha OS=Homo sapiens OX=9606 GN=TCP1 PE=1 SV=1 - [TCPA_HUMAN] | | 9.89 | 1 | 3 | 3 | 3 | 556 | 60.3 | 6.11 |
| 13 | pre-rRNA 2'-O-ribose RNA methyltransferase FTSJ3 OS=Homo sapiens OX=9606 GN=FTSJ3 PE=1 SV=2 - [SPB1_HUMAN] | | 9.09 | 1 | 3 | 3 | 4 | 847 | 96.5 | 8.40 |
| 14 | DNA replication licensing factor MCM4 OS=Homo sapiens OX=9606 GN=MCM4 PE=1 SV=5 - [MCM4_HUMAN] | | 5.91 | 1 | 3 | 3 | 3 | 863 | 96.5 | 6.74 |
| 15 | Sarcoplasmic/endoplasmic reticulum calcium ATPase 2 OS=Homo sapiens OX=9606 GN=ATP2A2 PE=1 SV=1 - [AT2A2_HUMAN] | | 5.18 | 3 | 3 | 3 | 3 | 1042 | 114.7 | 5.34 |
| 16 | Probable ATP-dependent RNA helicase DDX46 OS=Homo sapiens OX=9606 GN=DDX46 PE=1 SV=2 - [DDX46_HUMAN] | | 4.66 | 1 | 3 | 3 | 3 | 1031 | 117.3 | 9.29 |
| 17 | 40S ribosomal protein S15a OS=Homo sapiens OX=9606 GN=RPS15A PE=1 SV=2 - [RS15A_HUMAN] | | 26.15 | 1 | 2 | 2 | 2 | 130 | 14.8 | 10.13 |
| 18 | General transcription factor IIF subunit 2 OS=Homo sapiens OX=9606 GN=GTF2F2 PE=1 SV=2 - [T2FB_HUMAN] | | 20.88 | 1 | 2 | 2 | 2 | 249 | 28.4 | 9.23 |
| 19 | Prohibitin OS=Homo sapiens OX=9606 GN=PHB PE=1 SV=1 - [PHB_HUMAN] | | 12.50 | 1 | 2 | 2 | 3 | 272 | 29.8 | 5.76 |
| 20 | rRNA 2'-O-methyltransferase fibrillarin OS=Homo sapiens OX=9606 GN=FBL PE=1 SV=2 - [FBRL_HUMAN] | | 9.35 | 2 | 2 | 2 | 2 | 321 | 33.8 | 10.18 |
| 21 | Pre-mRNA-splicing factor SYF1 OS=Homo sapiens OX=9606 GN=XAB2 PE=1 SV=2 - [SYF1_HUMAN] | | 5.26 | 1 | 2 | 2 | 2 | 855 | 99.9 | 6.23 |
| 22 | Lymphoid-specific helicase OS=Homo sapiens OX=9606 GN=HELLS PE=1 SV=1 - [HELLS_HUMAN] | | 4.65 | 1 | 2 | 2 | 2 | 838 | 97.0 | 7.93 |
| 23 | Synapsin-2 OS=Homo sapiens OX=9606 GN=SYN2 PE=2 SV=4 - [SYN2_HUMAN] | | 4.64 | 1 | 2 | 2 | 2 | 582 | 63.0 | 8.41 |
| 24 | Phosphate carrier protein, mitochondrial OS=Homo sapiens OX=9606 GN=SLC25A3 PE=1 SV=2 - [MPCP_HUMAN] | | 4.42 | 1 | 2 | 2 | 2 | 362 | 40.1 | 9.38 |
| 25 | DNA replication licensing factor MCM7 OS=Homo sapiens OX=9606 GN=MCM7 PE=1 SV=4 - [MCM7_HUMAN] | | 4.31 | 1 | 2 | 2 | 2 | 719 | 81.3 | 6.46 |
| 26 | Delta-1-pyrroline-5-carboxylate synthase OS=Homo sapiens OX=9606 GN=ALDH18A1 PE=1 SV=2 - [P5CS_HUMAN] | | 3.65 | 1 | 2 | 2 | 3 | 795 | 87.2 | 7.12 |
| 27 | AP-3 complex subunit delta-1 OS=Homo sapiens OX=9606 GN=AP3D1 PE=1 SV=1 - [AP3D1_HUMAN] | | 3.21 | 1 | 2 | 2 | 2 | 1153 | 130.1 | 8.48 |
| 28 | Nuclear pore complex protein Nup214 OS=Homo sapiens OX=9606 GN=NUP214 PE=1 SV=2 - [NU214_HUMAN] | | 2.97 | 1 | 2 | 2 | 2 | 2090 | 213.5 | 7.47 |
| 29 | Bifunctional glutamate/proline--tRNA ligase OS=Homo sapiens OX=9606 GN=EPRS PE=1 SV=5 - [SYEP_HUMAN] | | 2.78 | 1 | 2 | 2 | 2 | 1512 | 170.5 | 7.33 |
| 30 | DNA mismatch repair protein Msh6 OS=Homo sapiens OX=9606 GN=MSH6 PE=1 SV=2 - [MSH6_HUMAN] | | 2.72 | 1 | 2 | 2 | 2 | 1360 | 152.7 | 6.90 |
| 31 | Cytoplasmic dynein 1 heavy chain 1 OS=Homo sapiens OX=9606 GN=DYNC1H1 PE=1 SV=5 - [DYHC1_HUMAN] | | 1.36 | 1 | 2 | 2 | 3 | 4646 | 532.1 | 6.40 |
| 32 | Ankyrin repeat domain-containing protein 17 OS=Homo sapiens OX=9606 GN=ANKRD17 PE=1 SV=3 - [ANR17_HUMAN] | | 1.04 | 2 | 2 | 2 | 2 | 2603 | 274.1 | 6.52 |
| 33 | Pro-cathepsin H OS=Homo sapiens OX=9606 GN=CTSH PE=1 SV=4 - [CATH_HUMAN] | | 12.84 | 1 | 1 | 1 | 1 | 335 | 37.4 | 8.07 |
| 34 | Protein CREG2 OS=Homo sapiens OX=9606 GN=CREG2 PE=1 SV=1 - [CREG2_HUMAN] | | 12.76 | 1 | 1 | 1 | 1 | 290 | 32.1 | 8.98 |
| 35 | 60S ribosomal protein L23 OS=Homo sapiens OX=9606 GN=RPL23 PE=1 SV=1 - [RL23_HUMAN] | | 10.71 | 1 | 1 | 1 | 1 | 140 | 14.9 | 10.51 |
| 36 | Cytochrome c OS=Homo sapiens OX=9606 GN=CYCS PE=1 SV=2 - [CYC_HUMAN] | | 10.48 | 1 | 1 | 1 | 1 | 105 | 11.7 | 9.57 |
| 37 | U3 small nucleolar ribonucleoprotein protein IMP3 OS=Homo sapiens OX=9606 GN=IMP3 PE=1 SV=1 - [IMP3_HUMAN] | | 9.78 | 1 | 1 | 1 | 1 | 184 | 21.8 | 9.50 |
| 38 | ER lumen protein-retaining receptor 1 OS=Homo sapiens OX=9606 GN=KDELR1 PE=1 SV=1 - [ERD21_HUMAN] | | 8.96 | 1 | 1 | 1 | 1 | 212 | 24.5 | 8.62 |
| 39 | Saccharopine dehydrogenase-like oxidoreductase OS=Homo sapiens OX=9606 GN=SCCPDH PE=1 SV=1 - [SCPDL_HUMAN] | | 6.53 | 1 | 1 | 1 | 2 | 429 | 47.1 | 9.14 |
| 40 | 40S ribosomal protein S6 OS=Homo sapiens OX=9606 GN=RPS6 PE=1 SV=1 - [RS6_HUMAN] | | 6.02 | 1 | 1 | 1 | 1 | 249 | 28.7 | 10.84 |
| 41 | 60S ribosomal protein L5 OS=Homo sapiens OX=9606 GN=RPL5 PE=1 SV=3 - [RL5_HUMAN] | | 5.72 | 1 | 1 | 1 | 1 | 297 | 34.3 | 9.72 |
| 42 | Activator of 90 kDa heat shock protein ATPase homolog 1 OS=Homo sapiens OX=9606 GN=AHSA1 PE=1 SV=1 - [AHSA1_HUMAN] | | 5.62 | 1 | 1 | 1 | 1 | 338 | 38.3 | 5.53 |
| 43 | G patch domain-containing protein 4 OS=Homo sapiens OX=9606 GN=GPATCH4 PE=1 SV=2 - [GPTC4_HUMAN] | | 5.38 | 1 | 1 | 1 | 1 | 446 | 50.4 | 9.63 |
| 44 | Polycomb group RING finger protein 6 OS=Homo sapiens OX=9606 GN=PCGF6 PE=1 SV=2 - [PCGF6_HUMAN] | | 4.57 | 1 | 1 | 1 | 1 | 350 | 39.0 | 4.97 |
| 45 | X-ray repair cross-complementing protein 5 OS=Homo sapiens OX=9606 GN=XRCC5 PE=1 SV=3 - [XRCC5_HUMAN] | | 4.23 | 1 | 1 | 1 | 1 | 732 | 82.7 | 5.81 |
| 46 | Trichoplein keratin filament-binding protein OS=Homo sapiens OX=9606 GN=TCHP PE=1 SV=1 - [TCHP_HUMAN] | | 4.22 | 1 | 1 | 1 | 1 | 498 | 61.0 | 6.54 |
| 47 | U4/U6 small nuclear ribonucleoprotein Prp31 OS=Homo sapiens OX=9606 GN=PRPF31 PE=1 SV=2 - [PRP31_HUMAN] | | 4.21 | 1 | 1 | 1 | 1 | 499 | 55.4 | 5.78 |
| 48 | Elongation factor Tu, mitochondrial OS=Homo sapiens OX=9606 GN=TUFM PE=1 SV=2 - [EFTU_HUMAN] | | 3.98 | 1 | 1 | 1 | 2 | 452 | 49.5 | 7.61 |
| 49 | E3 ubiquitin/ISG15 ligase TRIM25 OS=Homo sapiens OX=9606 GN=TRIM25 PE=1 SV=2 - [TRI25_HUMAN] | | 3.97 | 1 | 1 | 1 | 1 | 630 | 70.9 | 8.09 |
| 50 | ATP-dependent RNA helicase DDX3Y OS=Homo sapiens OX=9606 GN=DDX3Y PE=1 SV=2 - [DDX3Y_HUMAN] | | 3.64 | 2 | 1 | 1 | 1 | 660 | 73.1 | 7.55 |
| 51 | Zinc finger protein 281 OS=Homo sapiens OX=9606 GN=ZNF281 PE=1 SV=1 - [ZN281_HUMAN] | | 3.35 | 1 | 1 | 1 | 1 | 895 | 96.9 | 8.48 |
| 52 | Aspartate aminotransferase, mitochondrial OS=Homo sapiens OX=9606 GN=GOT2 PE=1 SV=3 - [AATM_HUMAN] | | 3.26 | 1 | 1 | 1 | 1 | 430 | 47.5 | 9.01 |
| 53 | Glycogen phosphorylase, brain form OS=Homo sapiens OX=9606 GN=PYGB PE=1 SV=5 - [PYGB_HUMAN] | | 2.97 | 1 | 1 | 1 | 1 | 843 | 96.6 | 6.86 |
| 54 | Nucleolar GTP-binding protein 1 OS=Homo sapiens OX=9606 GN=GTPBP4 PE=1 SV=3 - [NOG1_HUMAN] | | 2.84 | 1 | 1 | 1 | 1 | 634 | 73.9 | 9.50 |
| 55 | Ras-related protein Rab-3D OS=Homo sapiens OX=9606 GN=RAB3D PE=1 SV=1 - [RAB3D_HUMAN] | | 2.74 | 4 | 1 | 1 | 1 | 219 | 24.3 | 4.93 |
| 56 | Interferon-induced, double-stranded RNA-activated protein kinase OS=Homo sapiens OX=9606 GN=EIF2AK2 PE=1 SV=2 - [E2AK2_HUMAN] | | 2.72 | 1 | 1 | 1 | 1 | 551 | 62.1 | 8.40 |
| 57 | Ras/Rap GTPase-activating protein SynGAP OS=Homo sapiens OX=9606 GN=SYNGAP1 PE=1 SV=4 - [SYGP1_HUMAN] | | 2.68 | 1 | 1 | 1 | 1 | 1343 | 148.2 | 8.98 |
| 58 | Programmed cell death 6-interacting protein OS=Homo sapiens OX=9606 GN=PDCD6IP PE=1 SV=1 - [PDC6I_HUMAN] | | 2.65 | 1 | 1 | 1 | 1 | 868 | 96.0 | 6.52 |
| 59 | Epiplakin OS=Homo sapiens OX=9606 GN=EPPK1 PE=1 SV=3 - [EPIPL_HUMAN] | | 2.61 | 1 | 1 | 2 | 4 | 5088 | 555.3 | 5.62 |
| 60 | Nucleolar protein 56 OS=Homo sapiens OX=9606 GN=NOP56 PE=1 SV=4 - [NOP56_HUMAN] | | 2.53 | 1 | 1 | 1 | 1 | 594 | 66.0 | 9.19 |
| 61 | Calcium/calmodulin-dependent protein kinase type II subunit alpha OS=Homo sapiens OX=9606 GN=CAMK2A PE=1 SV=2 - [KCC2A_HUMAN] | | 2.51 | 1 | 1 | 1 | 1 | 478 | 54.1 | 7.08 |
| 62 | Plakophilin-3 OS=Homo sapiens OX=9606 GN=PKP3 PE=1 SV=1 - [PKP3_HUMAN] | | 2.51 | 1 | 1 | 1 | 1 | 797 | 87.0 | 9.32 |
| 63 | Chromosome alignment-maintaining phosphoprotein 1 OS=Homo sapiens OX=9606 GN=CHAMP1 PE=1 SV=2 - [CHAP1_HUMAN] | | 2.46 | 1 | 1 | 1 | 1 | 812 | 89.0 | 8.44 |
| 64 | Deoxynucleotidyltransferase terminal-interacting protein 2 OS=Homo sapiens OX=9606 GN=DNTTIP2 PE=1 SV=2 - [TDIF2_HUMAN] | | 2.38 | 1 | 1 | 1 | 1 | 756 | 84.4 | 6.16 |
| 65 | U2 snRNP-associated SURP motif-containing protein OS=Homo sapiens OX=9606 GN=U2SURP PE=1 SV=2 - [SR140_HUMAN] | | 2.24 | 1 | 1 | 1 | 1 | 1029 | 118.2 | 8.47 |
| 66 | Nucleolar GTP-binding protein 2 OS=Homo sapiens OX=9606 GN=GNL2 PE=1 SV=1 - [NOG2_HUMAN] | | 2.19 | 1 | 1 | 1 | 1 | 731 | 83.6 | 9.25 |
| 67 | Nucleolar complex protein 2 homolog OS=Homo sapiens OX=9606 GN=NOC2L PE=1 SV=4 - [NOC2L_HUMAN] | | 2.14 | 1 | 1 | 1 | 1 | 749 | 84.9 | 5.62 |
| 68 | Elongin-A OS=Homo sapiens OX=9606 GN=ELOA PE=1 SV=2 - [ELOA1_HUMAN] | | 2.13 | 1 | 1 | 1 | 1 | 798 | 89.9 | 9.57 |
| 69 | Tropomodulin-2 OS=Homo sapiens OX=9606 GN=TMOD2 PE=1 SV=1 - [TMOD2_HUMAN] | | 1.99 | 1 | 1 | 1 | 1 | 351 | 39.6 | 5.27 |
| 70 | Dynamin-1 OS=Homo sapiens OX=9606 GN=DNM1 PE=1 SV=2 - [DYN1_HUMAN] | | 1.97 | 1 | 1 | 1 | 1 | 864 | 97.3 | 7.17 |
| 71 | Exosome component 10 OS=Homo sapiens OX=9606 GN=EXOSC10 PE=1 SV=2 - [EXOSX_HUMAN] | | 1.92 | 1 | 1 | 1 | 2 | 885 | 100.8 | 8.46 |
| 72 | SAFB-like transcription modulator OS=Homo sapiens OX=9606 GN=SLTM PE=1 SV=2 - [SLTM_HUMAN] | | 1.84 | 1 | 1 | 1 | 1 | 1034 | 117.1 | 7.87 |
| 73 | Heterochromatin protein 1-binding protein 3 OS=Homo sapiens OX=9606 GN=HP1BP3 PE=1 SV=1 - [HP1B3_HUMAN] | | 1.81 | 1 | 1 | 1 | 1 | 553 | 61.2 | 9.67 |
| 74 | Acetyl-CoA carboxylase 2 OS=Homo sapiens OX=9606 GN=ACACB PE=1 SV=3 - [ACACB_HUMAN] | | 1.75 | 1 | 1 | 3 | 4 | 2458 | 276.4 | 6.49 |
| 75 | Nucleolar protein 14 OS=Homo sapiens OX=9606 GN=NOP14 PE=1 SV=3 - [NOP14_HUMAN] | | 1.63 | 1 | 1 | 1 | 1 | 857 | 97.6 | 7.58 |
| 76 | ATP-dependent RNA helicase DDX24 OS=Homo sapiens OX=9606 GN=DDX24 PE=1 SV=1 - [DDX24_HUMAN] | | 1.63 | 1 | 1 | 1 | 1 | 859 | 96.3 | 9.06 |
| 77 | ATP-dependent RNA helicase DDX42 OS=Homo sapiens OX=9606 GN=DDX42 PE=1 SV=1 - [DDX42_HUMAN] | | 1.60 | 1 | 1 | 1 | 1 | 938 | 102.9 | 7.02 |
| 78 | SURP and G-patch domain-containing protein 2 OS=Homo sapiens OX=9606 GN=SUGP2 PE=1 SV=2 - [SUGP2_HUMAN] | | 1.29 | 1 | 1 | 1 | 1 | 1082 | 120.1 | 7.28 |
| 79 | Apoptotic chromatin condensation inducer in the nucleus OS=Homo sapiens OX=9606 GN=ACIN1 PE=1 SV=2 - [ACINU_HUMAN] | | 1.27 | 1 | 1 | 1 | 2 | 1341 | 151.8 | 6.43 |
| 80 | Coatomer subunit beta OS=Homo sapiens OX=9606 GN=COPB1 PE=1 SV=3 - [COPB_HUMAN] | | 1.26 | 1 | 1 | 1 | 1 | 953 | 107.1 | 6.05 |
| 81 | Replication factor C subunit 1 OS=Homo sapiens OX=9606 GN=RFC1 PE=1 SV=4 - [RFC1_HUMAN] | | 1.05 | 1 | 1 | 1 | 1 | 1148 | 128.2 | 9.36 |
| 82 | Tyrosine-protein kinase BAZ1B OS=Homo sapiens OX=9606 GN=BAZ1B PE=1 SV=2 - [BAZ1B_HUMAN] | | 1.01 | 1 | 1 | 1 | 1 | 1483 | 170.8 | 8.48 |
| 83 | E3 ubiquitin-protein ligase TRIP12 OS=Homo sapiens OX=9606 GN=TRIP12 PE=1 SV=1 - [TRIPC_HUMAN] | | 1.00 | 1 | 1 | 1 | 1 | 1992 | 220.3 | 8.48 |
| 84 | Trinucleotide repeat-containing gene 6A protein OS=Homo sapiens OX=9606 GN=TNRC6A PE=1 SV=2 - [TNR6A_HUMAN] | | 0.97 | 1 | 1 | 1 | 1 | 1962 | 210.2 | 7.01 |
| 85 | Nuclear pore complex protein Nup98-Nup96 OS=Homo sapiens OX=9606 GN=NUP98 PE=1 SV=4 - [NUP98_HUMAN] | | 0.94 | 1 | 1 | 1 | 1 | 1817 | 197.5 | 6.40 |
| 86 | Probable helicase with zinc finger domain OS=Homo sapiens OX=9606 GN=HELZ PE=1 SV=2 - [HELZ_HUMAN] | | 0.93 | 1 | 1 | 1 | 1 | 1942 | 218.8 | 7.42 |
| 87 | Spectrin beta chain, non-erythrocytic 2 OS=Homo sapiens OX=9606 GN=SPTBN2 PE=1 SV=3 - [SPTN2_HUMAN] | | 0.79 | 1 | 1 | 1 | 1 | 2390 | 271.2 | 6.11 |
| 88 | Plectin OS=Homo sapiens OX=9606 GN=PLEC PE=1 SV=3 - [PLEC_HUMAN] | | 0.77 | 1 | 1 | 2 | 3 | 4684 | 531.5 | 5.96 |
| 89 | Neurogenic locus notch homolog protein 2 OS=Homo sapiens OX=9606 GN=NOTCH2 PE=1 SV=3 - [NOTC2_HUMAN] | | 0.73 | 1 | 1 | 1 | 1 | 2471 | 265.2 | 5.14 |

Supplementary Table S6: CYCS potential E3 ligase from Ubibrowse

| E3 | E3GENE | SUBGENE | HOMO | PFAM | GO | NET | MOTIF | SCORE |
| --- | --- | --- | --- | --- | --- | --- | --- | --- |
| P22681 | CBL | CYCS | 1 | 1 | 4.05 | 1.69 | 6.61 | 0.84 |
| Q9UNE7 | STUB1 | CYCS | 1 | 1 | 5.73 | 2.39 | 1 | 0.736 |
| Q9HCE7 | SMURF1 | CYCS | 1 | 1 | 5.73 | 1.84 | 1 | 0.736 |
| O95155 | UBE4B | CYCS | 1 | 1 | 5.73 | 1.77 | 1 | 0.732 |
| P29590 | PML | CYCS | 1 | 1 | 5.73 | 2.2 | 1 | 0.728 |
| Q05516 | ZBTB16 | CYCS | 1 | 1 | 4.05 | 1.84 | 1 | 0.705 |
| Q14139 | UBE4A | CYCS | 1 | 1 | 5.73 | 1.29 | 1 | 0.704 |
| Q99728 | BARD1 | CYCS | 1 | 1 | 4.05 | 1.77 | 1 | 0.702 |
| P43034 | PAFAH1B1 | CYCS | 1 | 1 | 4.05 | 1.77 | 1 | 0.702 |
| P51617 | IRAK1 | CYCS | 1 | 1 | 4.05 | 1.77 | 1 | 0.702 |
| P46934 | NEDD4 | CYCS | 1 | 1 | 4.05 | 1.77 | 1 | 0.702 |
| Q86TM6 | SYVN1 | CYCS | 1 | 1 | 2.33 | 1.44 | 2.12 | 0.701 |
| Q9ULV8 | CBLC | CYCS | 1 | 1 | 4.05 | 1.44 | 1 | 0.683 |
| O14512 | SOCS7 | CYCS | 1 | 1 | 4.05 | 1.44 | 1 | 0.683 |
| Q06587 | RING1 | CYCS | 1 | 1 | 4.05 | 1.44 | 1 | 0.683 |
| O75592 | MYCBP2 | CYCS | 1 | 1 | 4.05 | 1.44 | 1 | 0.683 |
| O75150 | RNF40 | CYCS | 1 | 1 | 4.05 | 1.44 | 1 | 0.683 |
| Q8TBB1 | LNX1 | CYCS | 1 | 1 | 4.05 | 1.44 | 1 | 0.683 |
| Q9UPN9 | TRIM33 | CYCS | 1 | 1 | 5.73 | 1 | 1 | 0.681 |
| O60315 | ZEB2 | CYCS | 1 | 1 | 5.73 | 1 | 1 | 0.681 |
| Q15542 | TAF5 | CYCS | 1 | 1 | 4.05 | 1.29 | 1 | 0.672 |
| P02511 | CRYAB | CYCS | 1 | 1 | 2.93 | 1.99 | 1 | 0.667 |
| Q13309 | SKP2 | CYCS | 1 | 1 | 2.33 | 1.77 | 1.06 | 0.655 |
| Q13489 | BIRC3 | CYCS | 1 | 1 | 2.33 | 2.39 | 1 | 0.653 |
| Q96CA5 | BIRC7 | CYCS | 1 | 1 | 2.33 | 2.39 | 1 | 0.653 |
| Q9C026 | TRIM9 | CYCS | 1 | 1 | 4.05 | 1 | 1.06 | 0.653 |
| Q9NZS9 | BFAR | CYCS | 1 | 1 | 2.33 | 2.3 | 1 | 0.649 |
| P21580 | TNFAIP3 | CYCS | 1 | 1 | 2.33 | 1.77 | 1 | 0.649 |
| Q8IUQ4 | SIAH1 | CYCS | 1 | 1 | 2.33 | 1.77 | 1 | 0.649 |
| Q9NS56 | TOPORS | CYCS | 1 | 1 | 2.33 | 1.77 | 1 | 0.649 |
| P51948 | MNAT1 | CYCS | 1 | 1 | 2.33 | 1.77 | 1 | 0.649 |
| O14543 | SOCS3 | CYCS | 1 | 1 | 2.33 | 1.77 | 1 | 0.649 |
| P40337 | VHL | CYCS | 1 | 1 | 2.33 | 1.77 | 1 | 0.649 |
| Q99732 | LITAF | CYCS | 1 | 1 | 4.05 | 1 | 1 | 0.647 |
| Q8N448 | LNX2 | CYCS | 1 | 1 | 4.05 | 1 | 1 | 0.647 |
| Q9BVA0 | KATNB1 | CYCS | 1 | 1 | 4.05 | 1 | 1 | 0.647 |
| Q86Y01 | DTX1 | CYCS | 1 | 1 | 4.05 | 1 | 1 | 0.647 |
| Q8TEL6 | TRPC4AP | CYCS | 1 | 1 | 4.05 | 1 | 1 | 0.647 |
| Q9C035 | TRIM5 | CYCS | 1 | 1 | 4.05 | 1 | 1 | 0.647 |
| Q9BZR9 | TRIM8 | CYCS | 1 | 1 | 4.05 | 1 | 1 | 0.647 |
| Q00987 | MDM2 | CYCS | 1 | 1 | 2.33 | 1.69 | 1 | 0.645 |
| P98170 | XIAP | CYCS | 1 | 1 | 2.33 | 2.2 | 1 | 0.645 |
| O15151 | MDM4 | CYCS | 1 | 1 | 2.33 | 1.69 | 1 | 0.645 |
| Q09472 | EP300 | CYCS | 1 | 1 | 2.33 | 1.69 | 1 | 0.645 |
| P11142 | HSPA8 | CYCS | 1 | 1 | 2.93 | 1.7 | 1 | 0.642 |
| Q13356 | PPIL2 | CYCS | 1 | 1 | 2.93 | 1.29 | 1 | 0.64 |
| P49792 | RANBP2 | CYCS | 1 | 1 | 2.93 | 1.29 | 1 | 0.64 |
| Q9UDY8 | MALT1 | CYCS | 1 | 1 | 2.33 | 1.44 | 1.06 | 0.634 |
| Q96P09 | BIRC8 | CYCS | 1 | 1 | 2.33 | 1.87 | 1 | 0.628 |
| Q9UBF6 | RNF7 | CYCS | 1 | 1 | 2.33 | 1.44 | 1 | 0.628 |
| P38398 | BRCA1 | CYCS | 1 | 1 | 2.33 | 1.44 | 1 | 0.628 |
| O00257 | CBX4 | CYCS | 1 | 1 | 2.33 | 1.44 | 1 | 0.628 |
| Q969K3 | RNF34 | CYCS | 1 | 1 | 2.33 | 1.29 | 1 | 0.617 |
| P51668 | UBE2D1 | CYCS | 1 | 1 | 2.33 | 1.29 | 1 | 0.617 |
| Q86WT6 | TRIM69 | CYCS | 1 | 1 | 2.33 | 1.29 | 1 | 0.617 |
| O43255 | SIAH2 | CYCS | 1 | 1 | 2.33 | 1.29 | 1 | 0.617 |
| P63244 | GNB2L1 | CYCS | 1 | 1 | 1.51 | 1.84 | 1 | 0.609 |
| O60260 | PARK2 | CYCS | 1 | 1 | 1.51 | 2.39 | 1 | 0.609 |
| Q66K89 | E4F1 | CYCS | 1 | 1 | 1.51 | 1.77 | 1 | 0.605 |
| Q92831 | KAT2B | CYCS | 1 | 1 | 1.51 | 1.77 | 1 | 0.605 |
| P53804 | TTC3 | CYCS | 1 | 1 | 1.25 | 1 | 2.12 | 0.604 |
| Q86YJ5 | 9-Mar | CYCS | 1 | 1 | 1.25 | 1 | 2.12 | 0.604 |
| Q15386 | UBE3C | CYCS | 1 | 1 | 1.25 | 1 | 2.12 | 0.604 |
