# Supplementary figures and images for: USP53 plays an antitumor role in hepatocellular carcinoma through deubiquitination of cytochrome c
Source: Oncogenesis. 2022 Jun 2;11(1):31. doi: 10.1038/s41389-022-00404-8 (PMC9163188; doi:10.1038/s41389-022-00404-8)

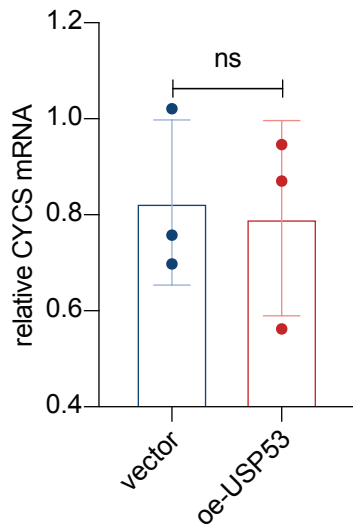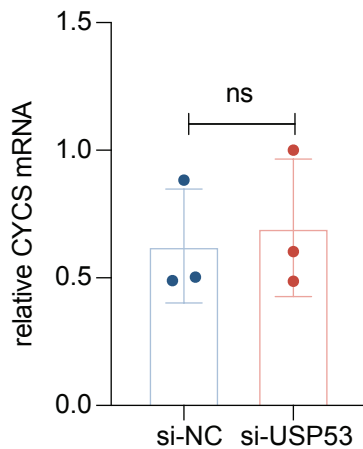

Supplement: Supplementary file 2 — supplementary figure1 [file 41389_2022_404_MOESM2_ESM.pdf]

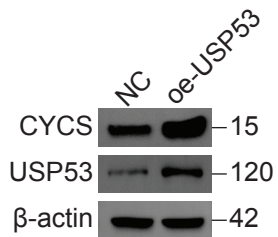

Supplement: Supplementary file 3 — supplementary figure2 [file 41389_2022_404_MOESM3_ESM.pdf]

**A**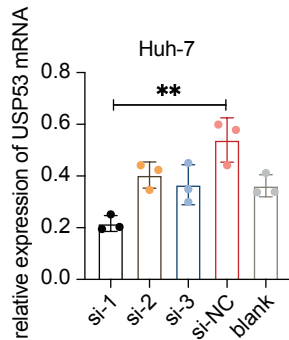**B**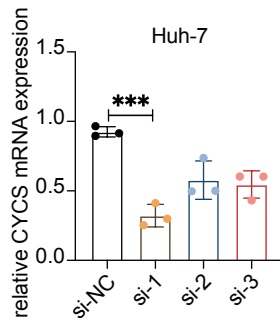

Supplement: Supplementary file 4 — supplementary figure3 [file 41389_2022_404_MOESM4_ESM.pdf]

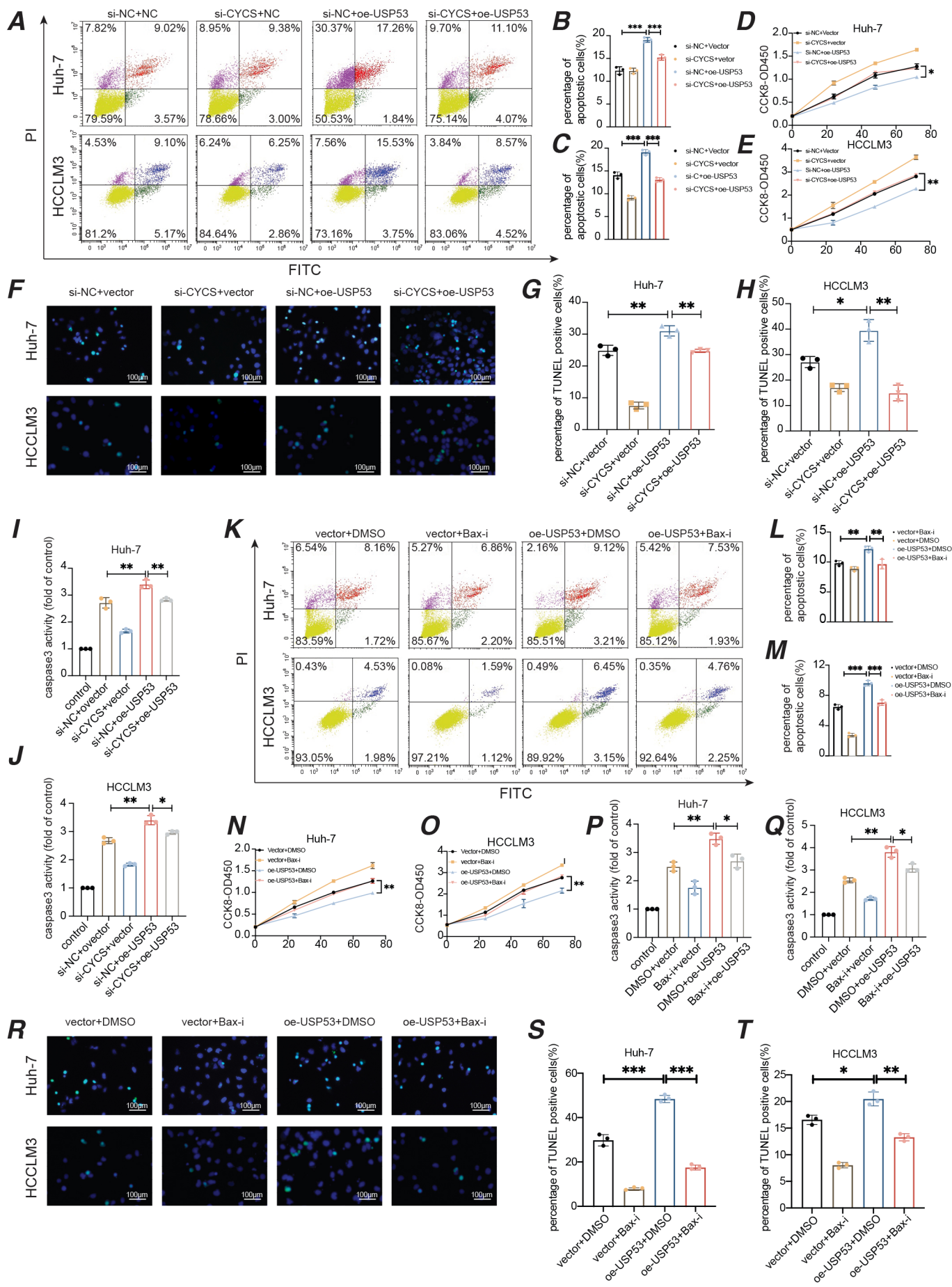

Supplement: Supplementary file 5 — supplementary figure4 [file 41389_2022_404_MOESM5_ESM.pdf]

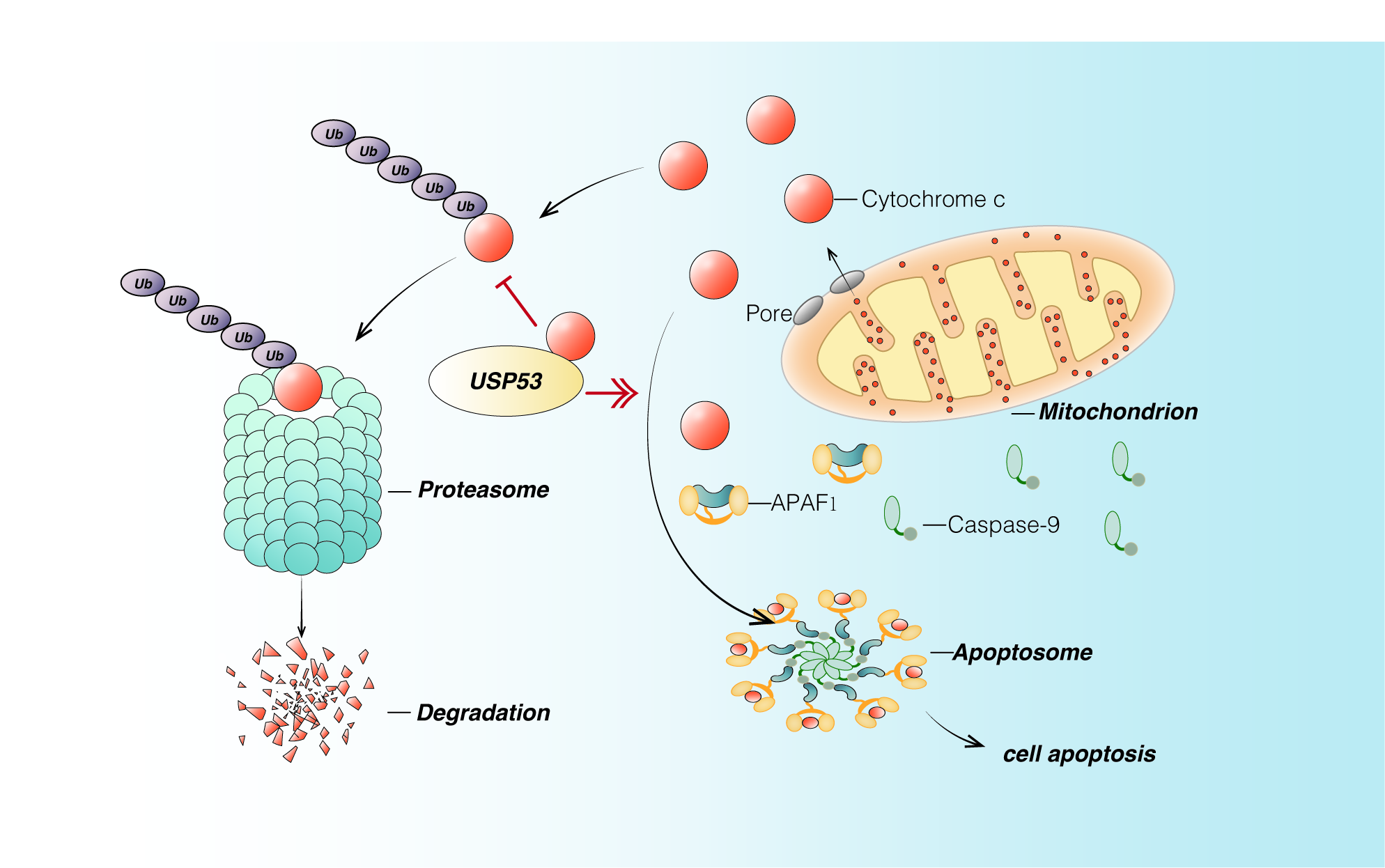

Supplement: Supplementary file 6 — supplementary figure5 [file 41389_2022_404_MOESM6_ESM.tif]

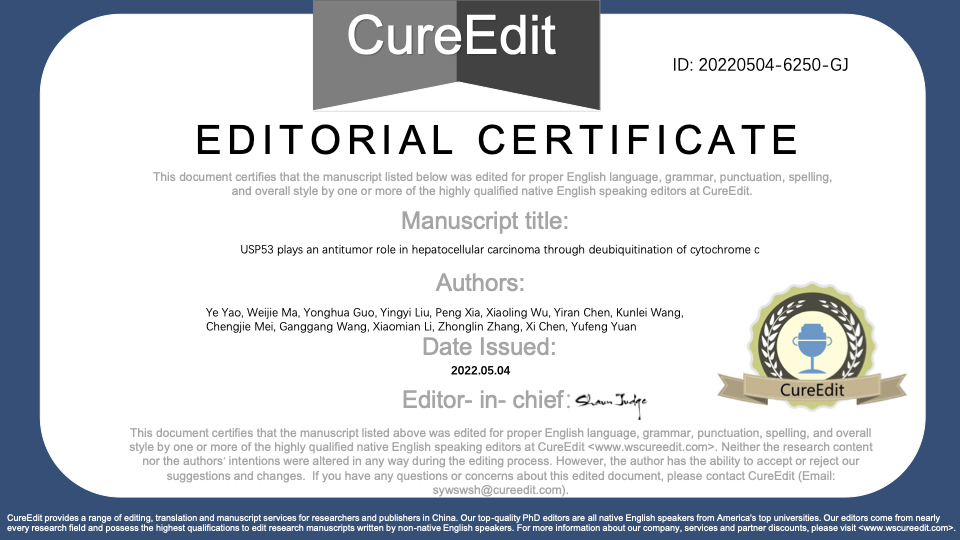

Supplement: Supplementary file 9 — language editorial certificate [file 41389_2022_404_MOESM9_ESM.tif]
