# Supplementary material for: USP53 plays an antitumor role in hepatocellular carcinoma through deubiquitination of cytochrome c
Source: Oncogenesis. 2022 Jun 2;11(1):31. doi: 10.1038/s41389-022-00404-8 (PMC9163188; doi:10.1038/s41389-022-00404-8)
Supplement: Supplementary file 7 — legend of supplementary figures [file 41389_2022_404_MOESM7_ESM.docx]

Supplementary Fig1. RT-qPCR analyses of CYCS mRNA in Huh-7 cells when USP53 was overexpressed or knocked down.

Supplementary Fig2. Western blotting of CYCS in xenografts of USP53 overexpressed tissue and control.

Supplementary Fig3. Validation of knockdown efficiency of siRNA. A. Relative expression of USP53 in USP53 knocked down cells and control. B. Relative expression of CYCS in CYCS knocked down cells and control. **p* < 0.05; ***p* < 0.01; ****p* < 0.001.

Supplementary Fig4. USP53 enhanced cell apoptosis when CYCS was inhibited. A-C. Flow cytometry plots showing the apoptotic rates in HCC cells when USP53 was overexpressed based on the inhibited BAX. BAX inhibitor was finally dissolved at a concentration of 50μM. n=3. D-E. CCK8 assay revealed the proliferative ability of the indicated groups. F-H. TUNEL assay revealed the apoptotic cells after USP53 overexpression in BAX-inhibited cells. Scale bar:100μm. I-K. Flow cytometry plots showing the apoptotic rates of HCC cells when USP53 was overexpressed in cells with CYCS knocked down. n=3. L-M. CCK8 assay revealed the proliferative ability of the indicated groups. N-P. TUNEL assay revealed the apoptotic percentage when USP53 was overexpressed in cells with CYCS knocked down. Scale bar:100μm. Q-T. Caspase-3 activity of the indicated groups of Huh-7 and HCCLM3 cells. Data are described with means±SD of the three independent experiments. Each value is expressed as the fold of caspase-3 activation level to the control level, and the value of control was set to 1. **p* < 0.05; ***p* < 0.01; ****p* < 0.001.

Supplementary Fig5. Schematic diagram of this study.
