# Supplementary material for: USP53 plays an antitumor role in hepatocellular carcinoma through deubiquitination of cytochrome c
Source: Oncogenesis. 2022 Jun 2;11(1):31. doi: 10.1038/s41389-022-00404-8 (PMC9163188; doi:10.1038/s41389-022-00404-8)
Supplement: Supplementary file 8 — statement for changing the order of author [file 41389_2022_404_MOESM8_ESM.pdf]

Changes to authorship

Reference: ONCSIS-21-0386RR

Article Entitled: USP53 plays an antitumor role in hepatocellular carcinoma through deubiquitination of cytochrome c

Authors: Ye Yao[1,2#], Weijie Ma[1,2#], Yonghua Guo[1,2#], Yingyi Liu[1,2], Peng Xia[1,2], Xiaoling Wu[1,2], Yiran Chen[1,2], Kunlei Wang[1,2], Chengjie Mei[1,2], Ganggang Wang[1,2], Xiaomian Li[1,2], Zhonglin Zhang[1,2], Xi Chen[1,2*], Yufeng Yuan[1,2*]

1 Department of Hepatobiliary and Pancreatic Surgery, Zhongnan Hospital of Wuhan University, Wuhan 430071 Hubei, China

2 Minimally invasive treatment center of hepatobiliary and pancreatic diseases, Zhongnan Hospital of Wuhan University, Wuhan 430071, Hubei, PR China.

[#] Ye Yao, Weijie Ma and Yonghua Guo contributes to this study equally.

*Correspondence Yufeng Yuan (yuanyf1971@whu.edu.cn) and Xi Chen(chenxi2022@whu.edu.cn), Department of Hepatobiliary and Pancreatic Surgery, Zhongnan Hospital of Wuhan University.

Dear editor,

Because of the author's contribution in the process of revision has changed, we need to change the current manuscript's author order and addresses, as listed above.

Authors' contributions

Conception and design: Y.Y, X.C, and Z.Z; Development of methodology: Y.Y, Y.L and W.M; Acquisition of data: Y.Y, X.C, Y.G, C.M, X.W; Analysis of data: Y.Y, X.C, Y.C, X.L, K.W, G.W; Writing, review and revision of the manuscript: Y.Y, Y.L, W.M, X.C, Y.G. All authors read and approved the final manuscript.

After consultations, all the authors agreed with the rearrangement of the names. In the final version of the article, Xi Chen is tagged as Co-corresponding author, Weijie Ma is tagged as Co-first author.

Below are the signatures of all authors.

Ye Yao Ye Yao

Weijie Ma Weijie Ma

Yonghua Guo Yonghua Guo

Yingyi Liu Yingyi Liu

Peng Xia Peng Xia

Xiaoling Wu Xiaoling Wu

Yiran Chen Yiran Chen

Kunlei Wang Wang Kunlei

Chengjie Mei Chengjie Mei

Ganggang Wang Ganggang Wang

Xiaomian Li Xiaomian Li

Zhonglin Zhang Zhonglin Zhang

Xi Chen Xi Chen

Yufeng Yuan Yufeng Yuan

Date: 5 April, 2022
